# Transmission dynamics of re-emerging rabies in domestic dogs of rural China

**DOI:** 10.1371/journal.ppat.1007392

**Published:** 2018-12-06

**Authors:** Huaiyu Tian, Yun Feng, Bram Vrancken, Bernard Cazelles, Hua Tan, Mandev S. Gill, Qiqi Yang, Yidan Li, Weihong Yang, Yuzhen Zhang, Yunzhi Zhang, Philippe Lemey, Oliver G. Pybus, Nils Chr. Stenseth, Hailin Zhang, Simon Dellicour

**Affiliations:** 1 State Key Laboratory of Remote Sensing Science, College of Global Change and Earth System Science, Beijing Normal University, Beijing, China; 2 Yunnan Institute of Endemic Diseases Control and Prevention, Yunnan Provincial Key Laboratory for Zoonosis Control and Prevention, Dali, China; 3 KU Leuven, Department of Microbiology and Immunology, Rega Institute, Laboratory of Evolutionary and Computational Virology, Leuven, Belgium; 4 Institut de Biologie de l’École Normale Supérieure UMR 8197, Eco-Evolutionary Mathematics, École Normale Supérieure, France; 5 Unité Mixte Internationnale 209, Mathematical and Computational Modeling of Complex Systems, Institut de Recherche pour le Développement et Université Pierre et Marie Curie, Bondy, France; 6 School of Biomedical Informatics, the University of Texas Health Science Center at Houston, Houston, Texas, United States of America; 7 Department of Zoology, University of Oxford, Oxford, United Kingdom; 8 Centre for Ecological and Evolutionary Synthesis (CEES), Department of Biosciences, University of Oslo, Blindern, Oslo, Norway; 9 Ministry of Education Key Laboratory for Earth System Modeling, Department of Earth System Science, Tsinghua University, Beijing, China; 10 Spatial Epidemiology Lab (SpELL), Université Libre de Bruxelles, Bruxelles, Belgium; Thomas Jefferson University, UNITED STATES

## Abstract

Despite ongoing efforts to control transmission, rabies prevention remains a challenge in many developing countries, especially in rural areas of China where re-emerging rabies is under-reported due to a lack of sustained animal surveillance. By taking advantage of detailed genomic and epidemiological data for the re-emerging rabies outbreak in Yunnan Province, China, collected between 1999 and 2015, we reconstruct the demographic and dispersal history of domestic dog rabies virus (RABV) as well as the dynamics of dog-to-dog and dog-to-human transmission. Phylogeographic analyses reveal a lower diffusion coefficient than previously estimated for dog RABV dissemination in northern Africa. Furthermore, epidemiological analyses reveal transmission rates between dogs, as well as between dogs and humans, lower than estimates for Africa. Finally, we show that reconstructed epidemic history of RABV among dogs and the dynamics of rabid dogs are consistent with the recorded human rabies cases. This work illustrates the benefits of combining phylogeographic and epidemic modelling approaches for uncovering the spatiotemporal dynamics of zoonotic diseases, with both approaches providing estimates of key epidemiological parameters.

## Author summary

Although dogs are known to be the primary reservoir and vector of human rabies in African and Asian countries, the spatial epidemiology of rabies virus (RABV) spread in developing regions is still unclear. Using 17 years of genomic and epidemiological data, we reconstruct the recent dispersal history of RABV in domestic dogs in Yunnan, a rural province of China, and estimate RABV transmission rate between dogs and from dogs to humans. Using a phylogeographic approach, we also evaluated the potential impact of several environmental factors on the mode and tempo of virus lineage dispersal. Our findings have implications for rabies prevention and control in Asian countries.

## Introduction

Rabies remains a significant threat to public health in the 21st century [1], causing around 60,000 human fatalities worldwide each year [2]. Rabies control in the developing world is currently hindered by a lack of timely and accurate data about rabies cases in both humans and animals [3]. It is thought that the number of human deaths due to rabies virus (RABV) infections is underestimated, and that the dynamics of the virus in dog populations is poorly understood. These uncertainties inevitably hamper improvements in disease control strategies and the evaluation of control measures.

China is second only to India [4] in terms of the national number of human rabies cases, and in recent years the prevalence of rabies has increased in some areas of China [5]. More than 90% of human rabies cases in China occur in rural regions [6] where the proportion of vaccinated dogs is very low [7]. Additionally, China has a growing population of dogs, currently estimated at 80–200 million animals [6], and the breeding, management, and vaccination of dogs in the country is uncontrolled [8]. A better quantitative understanding of rabies epidemiology in dogs is needed to help predict future vaccine demand in China and other developing countries.

Dogs are the primary reservoir and vector of human rabies throughout most of Africa and Asia [9] and are responsible for more than 99% of human rabies cases [1]. Therefore, understanding the dispersal dynamics of rabies in dogs is essential for quantifying dog-to-human transmissions and for disease prevention. Previous studies have documented “traveling waves” of rabies among wildlife populations [10–16] and uncovered the genetic signature of spatial expansion in RABV genomes [17–21]. Recent studies [22–24] have attempted to combine epidemiological and genomic data to study the transmission dynamics of RABV. However, rabies spread in rural areas of China, like Yunnan Province, remains poorly documented despite an increasing incidence of human rabies. Yunnan Province in Southwest China first reported human rabies in 1956 and eliminated the disease by integrated dog management and control measures (including dog registration and the use of dog enclosures, dog vaccination, and culling of rabid/suspected rabid dogs and stray dogs) in the late 1990s. However, a re-emergence of rabies in this province was reported in 1999.

**Fig 1:**
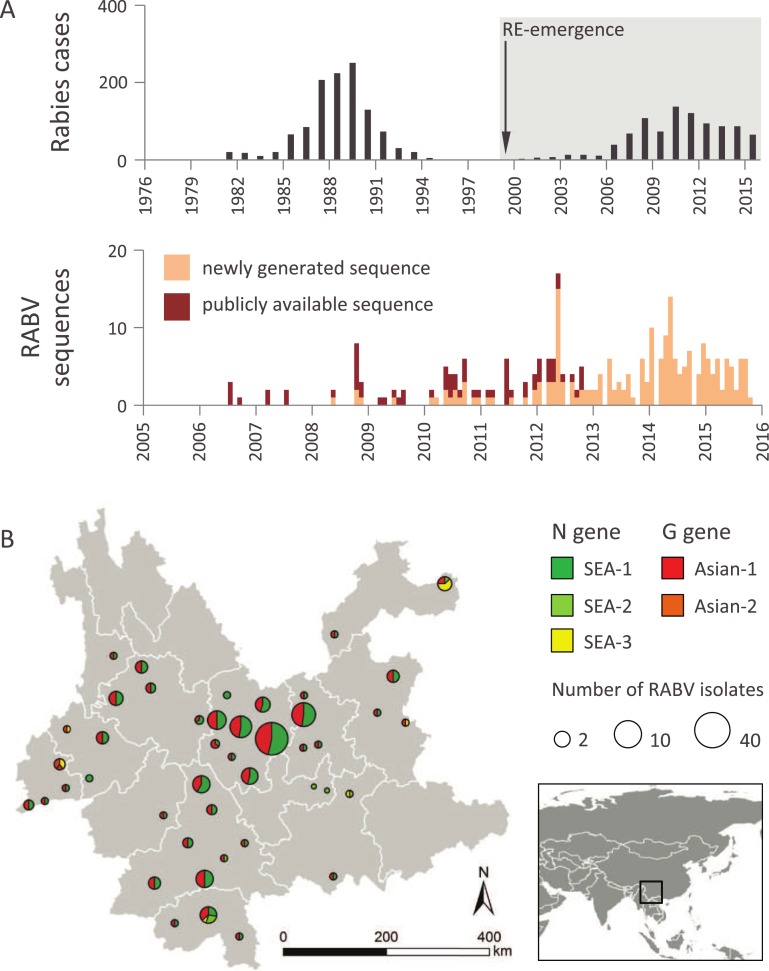
Current wave of rabies in Yunnan Province, China. **A.** Upper panel: human rabies in Yunnan Province from 1976 to 2015; lower panel: sampling of RABV samples in Yunnan Province. **B.** Locations of the RABV sequences sampled from dogs, 2006–2015. Circle areas are proportional to the number of gene-specific sequences sampled in each location and coloured according to a given clade (dark green: N gene clade SEA-1, green: N gene clade SEA-2, yellow: N gene clade SEA-3, red: G gene clade Asian-1, orange: G gene clade Asian-2).

In this study, we combine phylogeographic approaches and mathematical modelling to examine the dispersal dynamics of RABV in Yunnan, a rural province of China. We analyse publicly available and newly-generated RABV gene sequences from domestic dogs, cattle, and humans sampled between 2006 and 2015, together with comprehensive rabies epidemiological data dating back to 1999, when the first re-emerging human rabies case in Yunnan was reported (Fig 1A). Specifically, we aim to use both types of data to reconstruct the demographic and dispersal history of RABV spread in Yunnan, as well as to estimate key epidemiological parameters that can be compared to other previously-studied RABV outbreaks. Finally, we use phylogeographic inference to investigate which environmental factors may have impacted the spatial dispersal dynamics of RABV in Yunnan.

## Results

### Re-emergence of rabies in Yunnan

Analyses of case records date the first documented rabies case in Yunnan Province back to 1956. These data indicate that an epidemic wave of rabies occurred in the 1980s and that, from the mid-1990s onwards, human rabies cases were reported only sporadically in the region. However, starting in 1999, a new RABV epidemic emerged. More than 900 human rabies cases were reported during 1999–2015, with the highest incidence of 0.3 cases per 100,000 people occurring in 2010 (Fig 1A). Human rabies in Yunnan was first reported in 1999 in the Southeast prefecture of Wenshan, which borders Vietnam and the Chinese province of Guangxi and spread across the province in the next decade [25].

### Two RABV lineages dominate the Yunnan epidemic

**Fig 2:**
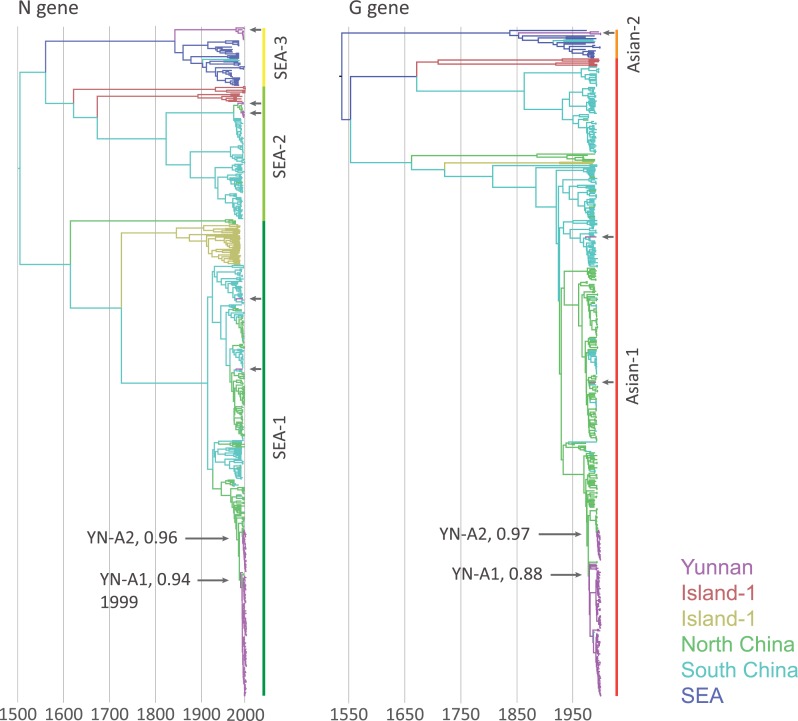
Maximum clade credibility trees of South-East Asian (SEA) RABV based on N and G gene sequences. Branches are coloured according to their most probable geographic location: Yunnan Province in China (magenta); Hebei, Beijing, Shanxi, Shaanxi, Ningxia, Shandong, Henan, Jiangsu, Anhui, Sichuan, Hubei and Chongqing in North China (green); Hunan, Jiangxi, Guizhou, Fujian, Guangxi, Shanghai and Zhejiang in South China (blue); China Taiwan and the Philippines (Island area-1, in red); Indonesia (Island-2, in yellow); Myanmar, Thailand, Laos, Vietnam and Cambodia in Southeast Asia (purple). The dominant lineages in Yunnan are labelled with YN-A1 and YN-A2, along with the posterior probabilities for their ancestral nodes. The grey arrows mark single Yunnan lineages while the black arrows point to Yunnan clusters.

We first used the discrete diffusion model implemented in BEAST 1.8 to undertake a continental-scale phylogeographic analysis of a broad data set of Asian RABV sequences (S1 Table; see also the Materials and Methods for details). Genetic histories of the RABV N and G genes reveal two individual lineages (YN-A1 and YN-A2, Fig 2) that represent the majority of sampled RABV infections in the post-1999 Yunnan epidemic.

We then used a GLM (generalised linear model) implementation, also available in BEAST 1.8, to measure the correlation between viral effective population size and RABV cases counts in Yunnan. This analysis estimated a GLM coefficient of 0.021 for the association between case counts and the trajectory of log effective population size of clade YN-A1 (which is the main RABV clade identified in Yunnan). The credible interval associated with this GLM coefficient estimate excludes zero (95% HPD: [0.005, 0.036]), and therefore indicates a significant association. The time to most recent common ancestor (tMRCA) estimates of the YN-A2 clade in the N and G gene largely overlap (N gene: 2007.8, 95% HPD: 2006.1–2009.2; G gene: 2008.3, 95% HPD: 2007.0–2009.4). The G gene estimate of the tMRCA of the YN-A1 lineage predates the N gene estimate (N gene: 1999.2, 95% HPD: 1996.6–2008.2; G gene: 1994.7, 95% HPD: 1992.5–1996.6).

### Expanding and more localised sub-epidemics

**Fig 3:**
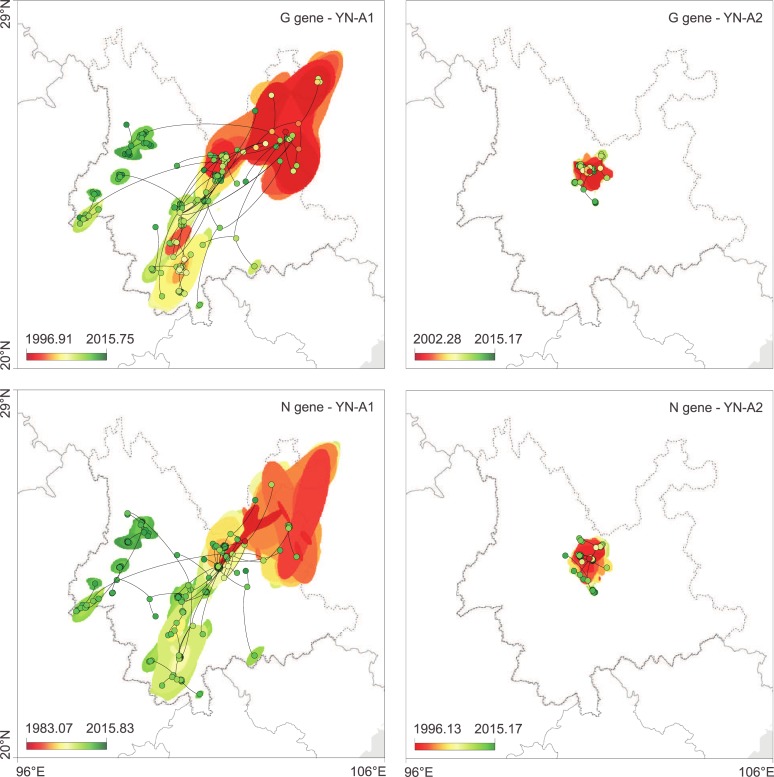
Spatiotemporal diffusion of Yunnan RABV clades estimated from continuous phylogeographic reconstructions. The plots show mapped trees and 95% HPD regions based on 100 trees subsampled from the post burn-in posterior distribution of trees. Nodes of the trees are coloured according to a colour scale ranging from red (the time to the most recent common ancestor, TMRCA) to green (most recent sampling time). 95% HPD regions were computed for successive time layers and then superimposed using the same colour scale reflecting time. National and Yunnan Province borders are shown by solid and dashed lines, respectively.

We next performed separate continuous phylogeographic reconstructions [26] for the YN-A1 and YN-A2 clades that were identified above in the discrete phylogeographic analysis. As shown in Fig 3, the inferred RABV diffusion histories estimated from the N and G gene alignments are consistent with each other. The YN-A1 clade underwent a larger spatial expansion and is more widely distributed within dog populations, while the YN-A2 clade appears to be geographically more limited. We compared the amino acid and nucleotide sites that vary between the YN-A1 and YN-A2 clades. There were no amino acid sites changes between these clades, suggesting that the differences in lineage distributions are not associated with pathobiological features, but rather over-representation of local area induced by stochastic founder events or by potential sampling bias (see Fig 1B).

Statistics of spatial dispersal estimated from continuous phylogeographic analyses are reported in Table 1. Estimation of diffusion coefficients [27] allows different outbreaks to be directly and quantitatively compared [19]. Specifically, we found that the diffusion coefficient of RABV in Yunnan (*D* = 1733 km^2^/year; 95% HPD = 1082–2928) is on average substantially lower than that previously estimated for RABV among North African dogs (*D* = 2874 km^2^/year; 95% HPD = 1900–5420) [28].

**Table 1:** RABV dispersion statistics estimated from continuous phylogeographic analyses.

Statistic	Definition	Median value	95% HPD
*v*_*branch*_	mean branch velocity	57.5 km/year	[39.2, 85.1]
*v*_*weighted*_	weighted dispersal velocity	23.4 km/year	[2.4, 32.6]
*D*_*original*_	original diffusion coefficient (Pybus *et al*. 2012)	1733 km^2^/year	[1082, 2928]
*D*_*weighted*_	weighted diffusion coefficient (Trovao *et al*. 2015)	1064 km^2^/year	[116, 1638]

### Impact of environmental factors on spatial dispersal dynamics

Several environmental variables were tested as potential factors that could affect RABV lineage dispersal velocity. As detailed in Materials and Methods, we estimated the correlation between phylogenetic branch durations and environmental distances computed on different rasters (S1 Fig). These correlations were then compared to the correlation between branch durations and distance computed on a “null” raster (i.e. an empty raster with no spatial heterogeneity whose cell values are set uniformly to “1”). Finally, the difference between these two correlations was assessed using a randomisation procedure. The rationale of this approach was to investigate which environmental factors can explain dispersal velocity heterogeneity better than simple geographic distances [28–30]. Complete results are reported in S2 Table, and S2 Fig reports results for the environmental factors that are most likely to have impacted virus lineage dispersal velocity. Only the forest coverage variable, tested as a potential conductance factor, was associated with a Bayes factor > 20. Although significant, the correlation is weak: using the “forest coverage” raster to compute environmental distances only increases the correlation with branch durations by 3%, relative to the uniform null raster. This means that forest coverage (treated as a conductance factor) would be a minor contributor to the observed heterogeneity in virus lineage dispersal velocity.

In addition to the analyses investigating the impact of environmental factors on dispersal *velocity*, we also investigated the impact of these factors on the dispersal *tendency*, i.e. we investigated if lineage translocation events tended to terminate in particular environmental conditions. Specifically, we compared environmental values at the beginning (oldest node) and end (youngest node) of each branch. Differences between the environmental values at each end of phylogeny branches tested using a randomisation procedure (see Materials and Methods). This analysis also indicated a possible effect of the “forest coverage” variable (Table 2): RABV lineages tended to spread towards areas associated with lower forest coverage (BF > 20 when considering forest coverage to be a potential negative driver of dispersal). We also note the relative importance of croplands (positive driver, BF = 17.2) and inaccessibility (negative driver, BF = 12.5) factors, meaning that the virus lineages were also more likely to spread towards croplands and accessible areas.

**Table 2:** Impact of several environmental factors on RABV lineage dispersal tendency in Yunnan. Each environmental varaiable was once tested as a positive and once as a negative driver of the viral dispersion. Bayes factors (BF) >3 and >20 can be considered as “positive” and “strong” evidences, respectively [13].

Environmental factor	BF when factors are treated as negative drivers	BF when factors are treated as positive drivers
Inaccessibility	12.5	0.1
Annual mean temperature	0.9	1.1
Annual precipitation	1.7	0.6
Croplands	0.1	17.2
Elevation	1.4	0.7
Foot print	0.1	7.1
Forests	21.2	0.0
Grasslands	0.3	3.2
Human pop. density	0.1	8.2
Savannas	1.3	0.8
Urban areas	0.1	6.6

### Dynamic modelling from epidemiological data and dog surveillance

**Fig 4:**
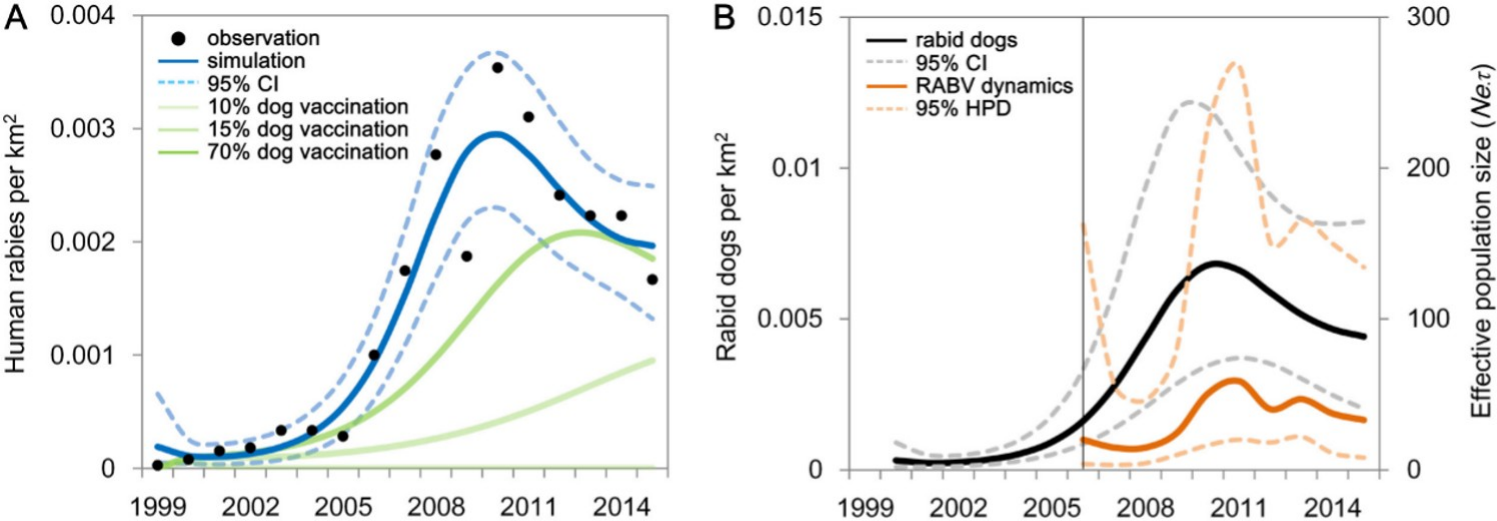
The current wave of human rabies in Yunnan Province from 1999 to 2015. (A) RABV transmission from dogs to humans. The black points represent observed human rabies cases. The blue line represents the deterministic prediction from the SEI model and the corresponding dotted light blue lines indicate the 95% credible interval endpoints of the model fit. Green lines show the simulation of intervention aimed at dogs with vaccination coverage of 10%, 15%, and 70% of dog population. (B) RABV transmission among dogs. The solid orange line is the posterior mean effective population size from the skygrid analysis inferred from the sequence data, and the surrounding dashed orange lines bound the 95% highest posterior density (HPD) region of that estimate. The solid black line is number of rabid dogs per square kilometre, and the dashed line is the 95% credible intervals. The vertical black line represents the earliest time point of sequence in study area.

We used mathematical transmission models and data from human and dog surveillance of RABV outbreaks in Yunnan Province to quantify rabies spread in a rural area of China. Dog demography and human rabies data were collected by the Yunnan Institute of Endemic Disease Control and Prevention. Our model-based simulation of dog-to-human RABV transmission captures the rapid increase in human rabies at the end of the year 2000, reaching a peak around 2010, before rapidly declining from 2011 onwards (Fig 4). Moreover, we find that the simulated density of rabid dogs is highly correlated with reconstruction of viral effective population size through time. Table 3 shows the fitted parameters and values used in the model. Trace plots as well as Gelman and Rubin diagnostic indicate convergence of the MCMC chains [31] (S3 Fig). The estimated carrying capacity (*K*) is 13.59 dogs/km^2^ [95% credible interval (CI): 7.80–19.37 dogs/km^2^]. The dog-to-dog transmission rate (*β*_*d*_) is 6.62/year, [95% CI: 5.60–7.64/year]. The dog-to-human transmission rate (*β*_*dh*_) is 0.0004/year, [95% CI: 0.0002–0.0006/year]. The average basic reproductive number (*R*_*0*_) of the most recent wave of infection is estimated at 1.05 during the early stages of the outbreak. High values for the transmission parameters and initial conditions are positively correlated with a high peak of human rabies infections (S4 Table), and the simulations are sensitive to these parameters. Our precise field measures are crucial for reliable model simulations, in particular the dog-to-human transmission rate and dog population size.

**Table 3:** RABV epidemiological parameters estimated from occurrence data.

Parameter	Definition	Mean value	SD*	
*b*_*d*_	dog birth rate	0.73/year	0.12	Estimated
*d*_*d*_	dog death rate	0.26/year	0.12	Estimated
*K*	dog carrying capacity	11.41 dogs/km^2^	0.83	Estimated
*σ*_*d*_	1/*σ*_*d*_ is the average latent period	13.43/year	1.82	Estimated
*α*_*d*_	death rate of rabid dogs	63.96/year	1.96	Estimated
*β*_*d*_	dog-to-dog transmission rate	6.62/year	0.52	Estimated
*β*_*dh*_	dog-to-human transmission rate	0.0004/year	0.0001	Estimated
*S*_0_	initial susceptible dog population size	9.50 dogs/km^2^	0.96	Estimated
*E*_0_	initial exposed dog population size	0.003 dogs/km^2^	0.003	Estimated
*I*_0_	initial infectious dog population size	0.003 dogs/km^2^	0.003	Estimated
*v*_*d*_	dog vaccination rate	0.07/year [32]	-	Fixed
*e*	dog vaccination success rate	0.60/year [32]	-	Fixed
*λ*_*d*_	dog loss of vaccination immunity rate	1/year [33]	-	Fixed
*λ*_*h*_	human loss of vaccination immunity rate	1/year	-	Fixed
*σ*_*h*_	1/*σ*_*h*_ is the average human incubation period	6/year [34]	-	Fixed
*α*_*h*_	rabid humans mortality rate	1/year	-	Fixed
*b*_*h*_	human birth rate	0.013/year [35]	-	Fixed
*d*_*h*_	human mortality rate	0.006/year [35]	-	Fixed

*standard deviation

## Discussion

Human rabies is one of the biggest public health risks facing China. While current rabies surveillance mostly reflects the RABV prevalence in humans, very little is known about its prevalence in dogs. In most of mainland China, diagnosis of dog rabies is currently effectively impossible in rural areas [8], because dogs are not leashed, can move freely and have a low vaccination coverage [6]. Because of this, rural dog populations are at increased risk of exposure and are crucial to rabies prevention and control efforts as they are the main reservoir for zoonoses [36]. Analysis of high-resolution epidemiological and genomic data provides an opportunity to explore the dynamics of re-emerging rabies dispersal and RABV transmission.

Continuous phylogeographic analyses yield a lower diffusion coefficient than that previously estimated for dog RABV in northern Africa [19,28]. Previous analysis of the northern Africa data set also revealed the importance of human-related environmental factors (human population density, accessibility to nearest major cities) as explanatory factors for the heterogeneity in RABV lineage dispersal velocity in Africa [28]. The higher diffusion coefficient of the northern African RABV data set was thus suspected to be related to human-based connectivity and/or mobility. Consequently, the lower diffusion coefficient estimated for the Yunnan data set could, in turn, indicate that human-related movements are less important contributors to the spatial dissemination of RABV in Yunnan, although we note that this is only one possible interpretation. Here, we also investigated the potential impact of several environmental factors and found a significant but weak effect of forest coverage acting (directly or indirectly) as a factor favouring RABV lineage dispersal velocity in Yunnan. One potential interpretation of this result is that in sparsely populated forest areas a few rapid (and potentially human-related) movements of infected dogs may have occurred. Such lineage dispersal movements could have happened via the road network crossing forest areas. However, even if identified as a significant conductance factor, the forest coverage in itself is not necessarily the causal factor and could instead be correlated with the true causal factor that is not included in the present analysis.

The potential importance of forest coverage was further indicated by comparing environmental conditions at branch termination locations. This analysis mainly revealed that viral lineages did not tend to spread towards forest areas. However, this result could also reflect, to some extent, the impact of a potential sampling bias arising if there were a lower sampling probability in less densely human-populated regions, such as forest areas. Furthermore, even under the assumption that viral lineages would be less likely to spread towards forest areas, our analysis based on dispersal velocity revealed that these areas do not act as barriers decreasing lineages dispersal velocity among infected areas.

Our dynamic model provides estimates of epidemiological parameters for dog rabies in Yunnan. The simulated dog population indicated a low level of RABV transmission, different from the oscillations observed in N’Djamena, Africa [11]. We also obtained an estimate of the transmission rate among dogs (*β*_*d*_), and from dogs to humans (*β*_*dh*_); key epidemiological parameters for assessment of rabies outbreaks and incidence among dogs. The estimate (*β*_*d*_) of 6.62/year was higher than the 4.20/year estimated in N’Djamena, Chad [34], and lower than 14.68/year estimated in Machakos District, Kenya [37,38]. The transmission rate from dogs to humans (*β*_*dh*_) was estimated to be 0.0004/year, which is consistent with our field investigation of dog bites (0.0004/year) in the study area [39], but much lower than the 0.0107/year estimated in N’Djamena [34]. In addition, the estimated carrying capacity (13.59 dogs/km^2^, 95% CI: 7.80–19.37 dogs/km^2^) was also lower than 33.6 dogs/km^2^ estimated in N’Djamena, Chad, but was similar to the value estimated in Tamil Nadu, India (12.69 dogs/km^2^, estimated using livestock census data) [40]. We suspect these may result from socio-economic differences, e.g. human population density [41].

Despite a national rabies control and prevention program that was implemented in China in 1985, which resulted in a drastic reduction in human rabies cases in the 1990s, a new RABV epidemic started in China in 1999 (Fig 1). Phylogeographic reconstructions confirm the East-to-West invasion history of RABV in Yunnan, which was previously suggested by epidemiological records [25]. Furthermore, we find a time lag between the peaks of viral effective population size and the corresponding peaks of rabid dog numbers (Fig 4). This may be due to the substantial proportion of undetected dog rabies infections; epidemiological surveillance of dog rabies in the region is far from exhaustive.

This study illustrates how RABV dispersion dynamics can be analysed by two complementary approaches based on genetic and epidemiological data. Overall, our work further highlights that domestic dogs play a key role in transmission and expansion of rabies in rural areas of China. The data reveal a low level of RABV transmission among dogs, and from dogs to humans, with the basic reproductive number estimated at 1.05 during the early stages of the outbreak. Our results indicate that interventions in the dog population would be effective in reducing transmission to humans, in particular because they have the potential to subvert the self-sustaining capacity of epidemics in dogs. A better understanding of RABV spread in terms of spatial and temporal dynamics is necessary to help inform the prevention and control of human rabies in the vast rural areas of China.

## Material and methods

### Ethics statement

It was determined by the Yunnan Institute of Endemic Diseases Control and Prevention, that the collection of data from rabies cases was part of continuing public health surveillance of a notifiable infectious disease and was exempt from institutional review board assessment. Experimental procedures were performed in compliance with guidelines established by the Chinese Center For Disease Control And Prevention and have been approved by ethics committee of Yunnan Institute of Endemic Diseases Control and Prevention.

### New samples from Yunnan

From 2008 to 2015, we collected 1392 brain tissue samples. These samples were obtained from 252 dogs suspected of having rabies, 1129 apparently healthy domestic dogs, 2 cows, and 9 human patients within 24 hours of death in 14 prefectures and 43 counties of Yunnan Province (Fig 1B). In addition, 18 saliva samples and 1 cerebrospinal fluid sample were obtained from surviving patients.

All brain specimens were tested using direct immunofluorescence assay (DFA) and RABV nucleoprotein monoclonal antibody (Rabies DFA Reagent; Chemicon, Temecula, CA, USA). Total RNA was extracted from the original brain samples with the Trizol reagent (Invitrogen, USA) according to the manufacturer’s instructions [25]. PCR products were purified by using a QIAquick PCR Purification Kit (QIAGEN, Germany). Complete nucleoprotein gene (N gene; 1 cow, 84 dog and 6 human isolates) and glycoprotein gene (G gene; 2 cow, 110 dog and 8 human isolates) sequences were obtained by using previously described primers [21,42]. Newly generated RABV sequence data were submitted to GenBank (accession numbers KP072009–KP072030, KP202418- KP202448, KT932670-KT932698 and KX096992-KX097000 for N gene, and JF819597-JF819602, JQ040570-JQ040581, JX276383-JX276404, KP072031-KP072052, KP202402-KP202417, KT861554-KT861586 and KX096983–KX096991 for G gene). An overview of the newly sequenced isolates is provided in S1 Table.

### Incidence data from the Yunnan RABV epidemic

Human rabies case records (Fig 1A) in Yunnan from 1999 to 2015 were obtained from the Yunnan Center for Disease Control and Prevention (CDC) and the Chinese CDC. In China, human rabies is a class B notifiable infectious disease and all human cases must be reported to the Chinese CDC. Rabies cases were confirmed according to diagnostic criteria (WS281–2008) from the Ministry of Health of the People’s Republic of China.

### Discrete phylogeographic analyses

To identify Yunnan-specific RABV circulation, nucleotide sequences of the N and G genes of all available RABV sequences sampled from non-flying mammals in Asia were downloaded from NCBI GenBank and aligned [43] together with our newly generated sequences. The resulting data sets included 543 N sequences (the entire N gene coding sequence, 1350 nt long) and 491 G sequences (the entire G gene coding sequence, 1575 nt long). RABV clades specific to Yunnan were identified using the discrete trait analysis model implemented in BEAST 1.8 [44,45]. For the molecular clock phylogeographic analyses, we specified a general time-reversible GTR+I nucleotide substitution model [46–48], a skygrid coalescent model [49] and a relaxed uncorrelated lognormal (UCLN) molecular clock model across branches [50]. The data sets lack a clear temporal signal [51] (S1 Fig). Therefore, we specified informative prior distributions on the gene-specific mean clock rate parameter. These prior distributions were based on previously published estimated substitution rates [52]: 1.88×10^−4^ substitutions/site/year (95% highest posterior density, HPD: [1.37×10^−4^, 2.41×10^−4^]) for the N gene and 2.13×10^−4^ substitutions/site/year (95% HPD: [1.56×10^−4^, 2.73×10^−4^]) for the G gene. The MCMC chain was run for 250 million states and mixing and convergence were inspected using Tracer [http://tree.bio.ed.ac.uk/software/tracer/]. TreeAnnotator 1.8 [44] was used to infer maximum clade credibility (MCC) summary trees.

### Testing covariates of viral population size

We used the GLM (generalised linear model) extension of the skygrid coalescent model [53], implemented in BEAST 1.8, to simultaneously infer viral effective population sizes and measure the association between estimated effective population size and cases counts. For this analysis, we focused only on sequences associated with the largest Yunnan clade identified by the discrete phylogeographic analysis (clade YN-A1, see the Results section). Furthermore, we considered the N and G genes as two independent markers (see Appendix S1 in S1 Text for the detailed procedure).

### Continuous phylogeographic analyses

The history of virus lineage dispersal in Yunnan was recovered from geo-referenced phylogenies, which were estimated using the continuous phylogeographic method [26] implemented in BEAST 1.8. A separate continuous phylogeographic analysis was performed for each gene and each Yunnan clade identified by the preliminary discrete phylogeographic analyses; however, the nucleotide substitution and molecular clock models were linked across the clades to avoid over-parameterisation. For these models, we used the same models as described above, together with a relaxed random walk model for inference of the continuous spatial locations. This relaxed random walk model assumed a log-normal probability distribution among phylogeny branches of diffusion rate scalars. The spatiotemporal information contained in inferred phylogenetic trees was extracted with the R package “seraphim” [29,30]. For the present study, we extracted spatiotemporal information from a subset of 1,000 trees sampled at regular intervals from the posterior distribution of trees (after burn-in had been removed). This was done for each gene and for each Yunnan clade. After this extraction step, each phylogeny branch is represented as a distinct movement vector [27]. “seraphim” was also used to estimate statistics of spatial dispersal based on these extracted movement vectors. We estimated the mean branch velocity, the weighted dispersal velocity, the diffusion coefficient (as originally defined in Pybus *et al*. [27]) and the weighted diffusion coefficient (as defined by Trovão *et al*. [54]). Further details regarding these statistics can be found in the “seraphim” package [30].

### Impact of environmental factors on spatial dispersal dynamics

We next sought to investigate the impact of environmental factors on lineage dispersal velocity. These analyses followed a similar structure to those used in previous studies [28,29,55]. All scripts for these analyses are available in the R package “seraphim” [30]. Here, we investigated the impact of the following environmental variables (S1 Fig): elevation, annual mean temperature, annual precipitation, key land cover variables (e.g. “grasslands”, “savannas”, “forests”, “croplands”, “urban areas”; land cover categorised according to the International Geosphere Biosphere Program, IGBP), human population density, human footprint, major roads, and inaccessibility (quantified as the time it takes to travel to the nearest major city of >50,000 inhabitants). The sources of the data in the original raster files are listed in S3 Table. All factors were tested as potential conductance factors (i.e. factors facilitating movement) and as potential resistance factors (i.e. factors impeding movement). Correlations between phylogenetic branch durations and environmentally-scaled distances were quantified as a statistic *Q*, which represents the difference between two coefficients of determination (R^2^): (i) the R^2^ obtained when branch durations are regressed against environmentally-scaled distances, and (ii) the R^2^ obtained when branch durations are regressed against distances computed on a “null” raster, i.e. a raster with a value of “1” assigned to every cell. An environmental factor was only considered as potentially explanatory if both its distribution of regression coefficients and its associated distribution of *Q* values were positive [56]. In a positive distribution of estimated *Q* values (i.e. with at least 90% of positive values), statistical support was then evaluated against a null distribution generated by a randomisation procedure and formalised using a Bayes factor (BF) value [28] (see Appendix S2 in S1 Text for the full procedure). Due to computational limits, this analysis was based on 100 trees subsampled from each post-burn-in posterior distribution obtained by continuous phylogeographic inference.

Addition to the analyses based on lineage dispersal *velocity*, we also used a new analytical procedure to investigate the impact of several environmental factors on dispersal *tendency*. In order words, this procedure aims at testing if virus lineages tend to disperse towards particular environmental conditions. In this framework, environmental conditions are compared between the locations of the two nodes connected by a phylogeny branch. For each branch and environmental factor, we computed the difference in raster cell values between the start (oldest node) location and at the end (youngest node) location. These differences were then averaged within each sampled tree and evaluated against a null distribution generated by the same randomisation procedure used for the analyses of the impact on dispersal velocity (see Appendix S3 in S1 Text for the detailed procedure).

### Dynamic modelling based on epidemiological data and dog surveillance

In the dynamic modelling part of this study, we extended the discrete susceptible-exposed-infectious (*SEI*) model for dog rabies [10,11,34]. Our discrete-time model framework was developed by dividing a closed dog population into four rabies classes, susceptible (*S*_*d*_), exposed (*E*_*d*_), and infectious (*I*_*d*_), and vaccinated (*V*_*d*_). We then extended the model for dog rabies to include dog-to-human RABV transmission. A similar modelling framework has been applied to quantitatively assess the dynamics of RABV transmission [34]. *S*_*h*_ is the number of people susceptible to disease, *P*_*h*_ is the annual vaccinated individuals in Yunnan (based on medical records from Chinese Center For Disease Control and Prevention), *E*_*h*_ and *V*_*h*_ represents the number of exposed humans and immunized humans during the time period, respectively.

$$S_{d,t+1}=S_{d,t}+b_{d}N_{d,t}+\lambda_{d}V_{d,t}-d_{d}S_{d,t}-\gamma N_{d,t}S_{d,t}-\beta_{d}S_{d,t}I_{d,t}-veS_{d,t}$$ [1]

$$E_{d,t+1}=E_{d,t}+\beta_{d}S_{d,t}I_{d,t}-d_{d}E_{d,t}-\gamma N_{d,t}E_{d,t}-\sigma E_{d,t}-veE_{d,t}$$ [2]

$$I_{d,t+1}=I_{d,t}+\sigma E_{d,t}-d_{d}I_{d,t}-\gamma N_{d,t}I_{d,t}-\alpha_{d}I_{d,t}$$ [3]

$$V_{d,t+1}=V_{d,t}+ve\left(S_{d,t}+E_{d,t}\right)-d_{d}V_{d,t}-\gamma N_{d,t}V_{d,t}-\lambda_{d}V_{d,t}$$ [4]

$$S_{h,t+1}=S_{h,t}\left(1-\beta_{dh}I_{d,t}\right)+b_{h}\left(S_{h,t}+E_{h,t}+V_{h,t}\right)-d_{h}S_{h,t}+\lambda_{h}V_{h,t}-P_{h,t}$$ [5]

$$E_{h,t+1}=\beta_{dh}S_{h,t}I_{d,t}-\sigma_{h}E_{h,t}-d_{h}E_{h,t}$$ [6]

$$I_{h,t+1}=\sigma_{h}E_{h,t}-d_{h}I_{h,t}-\alpha_{h}I_{h,t}$$ [7]

$$V_{h,t+1}=V_{h,t}\left(1-\lambda_{h}\right)+P_{h,t}-d_{h}V_{h,t}$$ [8]

where *N*_*d*_ is the total dog population (*S*_*d*_ + *E*_*d*_ + *I*_*d*_ + *V*_*d*_). Dog population growth is considered to be density-dependent, with birth (*b*_*d*_) and death (*d*_*d*_) rates. The density-dependent mortality parameter (*γ*) is a function of the dog population growth rate per year (*b*_*d*_—*d*_*d*_) and the carrying capacity density of the dog population in km^-2^ (*K*) is defined by *γ =* (*b*_*d*_—*d*_*d*_) / *K*. Carrying capacity is defined as the maximum number or density of individuals that can be sustainably supported by a given environment. In rural areas of China, many domestic dogs are free-roaming and there has been no population control for most of the time. We can then assume that dog populations (including owned and stray dogs) are regulated through represented by carrying capacity determined by some unknown combination of environmental and human geographic factors. *β*_*d*_ and *β*_*dh*_ are the dog-to-dog and dog-to-human transmission rate, respectively. *σ*_*d*_ is the latent-to-infectious rate per year and *α* is the rabies-induced mortality. At the beginning (*t* = 0), the basic reproductive number (*R*_*0*_) is *σβ*_d_*S*_0_/((*σ*+*b*)(*α*+*b*)). Informative prior distributions are assumed for death rate for rabid dog/human, latent period, incubation period, as well as dog-to-human transmission rate (*β*_*dh*_), which is assessed from our field investigation of dog bites [39]. We assumed dog vaccination rate (*v*) of 0.07 and dog vaccination success rate (*e*) of 0.60 in Yunnan by previous surveillance [32]. *λ* is loss of vaccination immunity rate. Culling was simulated assuming the elimination of 1% of the total dog population based on empirical estimation, due to the temporal irregular and changing intensity of potential culling. The susceptible reconstruction provided initial estimates of the initial susceptible dog population size, *S*_*d*,0_, for which we chose a uniform prior of 5–15 dogs/km^2^, according to our surveillance [32]. Initial numbers for *E*_*d*,0_ and *I*_*d*,0_ were unknown and fitted in the model with a wide prior 0–1 dogs/km^2^.

We fitted the dynamic model using the Bayesian state space framework using Metropolis-Hastings Markov chain Monte Carlo algorithms implemented in the MATLAB (vR2009b) toolbox DRAM (Delayed Rejection Adaptive Metropolis) [57,58]. The chain was initiated using published estimates of dog demography and RABV transmission rates [34,38,59–61] with an uninformative prior that varied from 0 to infinity, and then was run for 5 million iterations and sampled every 5000th step, after a burn-in of 500,000 iterations.

Sensitivity analysis was performed using latin hypercube sampling (LHS) and partial rank correlation coefficient (PRCC) techniques [62]. Two criteria were retained as outputs for the analysis: the intensity of the peak of the human rabies infections and the date of the peak. Uniform distributions were used for all parameters and the same ranges as for the prior distributions were used. A total of 1,000 parameter sets were sampled with LHS and PRCC was estimated using the MATLAB code provided by Marino et al. [63].

## Supporting information

S1 Text Appendix S1. Covariate analysis. Appendix S2. Analysis of the impact of environmental factor on RABV dispersal velocity in Yunnan. Appendix S3. Analysis of the impact of environmental factor on RABV dispersal tendency in Yunnan.
(DOCX)

S1 Fig Environmental variables that were tested in the analysis of the dog RABV dataset from Yunnan (China). National and Yunnan province borders are respectively displayed by solid and dashed lines, respectively.
(TIFF)

S2 Fig Potential impact of environmental factors on RABV dispersal velocity in Yunnan. The results are based on 100 trees sampled from the posterior distribution. “C” and “R” indicate if the considered environmental variable was considered as a conductance ("C") or resistance factor ("R"), and *k* is the rescaling parameter used to transform the initial raster. Estimated *Q* distributions and related Bayes factor (BF) supports are reported here only for those model combinations with >90% of positive *Q* values and a BF support > 3 (see S3 Table for complete results). BF supports were estimated with the randomisation procedure detailed in Appendix S1 in S1 Text. Following Kass & Raftery (1995) we consider a BF > 3 and BF >20 respectively as a “positive” and “strong” evidences of the statistical significance of *Q*, i.e. the correlation between environmental distances and dispersal durations (see text for further details).
(TIF)

S3 Fig Trace plots of four chains for each of the parameters. Four chains were initialized at different values, and they converge to the target distribution quickly.
(TIF)

S4 Fig Plots of the root-to-tip genetic distance against sampling time for N gene and G gene data sets of Yunnan. TempEst was used to obtain exploratory regressions based on the maximum likelihood trees.
(TIF)

S1 Table List of RABV sequences analysed in this study. (DOCX)

S2 Table Impact of several environmental factors on RABV dispersal velocity. The results are based on 100 trees sampled in each posterior distribution. “C” and “R” indicate if the considered environmental raster was considered as a conductance ("C") or resistance factor ("R"), and *k* is the rescaling parameter used to transform the initial raster (see the Appendix S2 in S1 Text for further details). For regression coefficients and *Q* values we report both the median estimate and the 95% HPD interval. The Bayes factor (BF) supports based on the randomisation procedure is only reported when *p*(*Q* > 0) is at least 90%. Following Kass & Raftery (1995) we consider a Bayes factor (BF) >3 as positive support for a significant correlation between the environmental distances and dispersal durations.
(DOCX)

S3 Table Source of data for each environmental raster. (DOCX)

S4 Table Sensitivity analysis. For model parameters, we used the same range as for the prior distribution. 1000 parameter sets were sampled with latin hypercube sampling, and partial rank correlation coefficients were estimated.
(DOCX)
