# Supplementary material for: Transmission dynamics of re-emerging rabies in domestic dogs of rural China
Source: PLoS Pathog. 2018 Dec 6;14(12):e1007392. doi: 10.1371/journal.ppat.1007392 (PMC6283347; doi:10.1371/journal.ppat.1007392)
Supplement: S1 Text — Appendix S1. Covariate analysis. Appendix S2. Analysis of the impact of environmental factor on RABV dispersal velocity in Yunnan. Appendix S3. Analysis of the impact of environmental factor on RABV dispersal tendency in Yunnan. (DOCX) [file ppat.1007392.s001.docx]

**S1_Text.**

**Appendix S1. Covariate analysis**

We used the GLM (generalised linear model) extension of the skygrid coalescent model, implemented in BEAST 1.8 [[12](#_ENREF_12)], to simultaneously infer viral effective population sizes and measure the association between estimated effective population size and cases counts. In this approach, the number of rabies cases through time was modelled as a covariate of viral effective population size, hence the effective population size was estimated by combining information from both the sequence and covariate data. For this analysis, we focused only on sequences associated with the largest Yunnan clade identified by the discrete phylogeographic analysis (clade YN-A1, see the Results section). Furthermore, we considered the N and G genes as two independent markers. For comparison, we also performed a skygrid reconstruction of the effective population size using only sequence data (i.e. without using the case report numbers as a covariate).

**Appendix S2. Analysis of the impact of environmental factor on RABV dispersal velocity in Yunnan**

*Step 1: extraction of spatio-temporal information contained in phylogenetic trees.*

We selected a subset of 100 trees sampled at regular intervals from the posterior distribution of trees (after burn-in had been removed) inferred for each clade. The spatio-temporal information embedded in these trees was extracted with the “treeExtractions” function of the R package “seraphim” [[1](#_ENREF_1)]. Specifically, each phylogenetic branch was considered a vector defined by its start and end location (latitude and longitude), and its start and end dates (in decimal units). Each phylogeny branch therefore represents a conditionally independent viral lineage dispersal event [[2](#_ENREF_2)]. In the specific context of this study, we decided to gather all movement vectors extracted from the different Yunnan clades for subsequent analyses.

*Step 2: computing environmental distances associated with phylogeny branches.*

All the vectors obtained in step 1 were assigned an “environmental distance”, i.e. a spatial distance that was weighted according to the values of an environmental raster at each location [[3](#_ENREF_3)]. We used two distinct path models to compute the environmental distance allocated to each phylogeny branch for a given environmental factor: (i) the least-cost path model, which uses a least-cost algorithm to determine the route taken between the start and end points [[4](#_ENREF_4)], and (ii) the Circuitscape path model, which uses circuit theory to accommodate uncertainty in the route taken [[5](#_ENREF_5), [6](#_ENREF_6)]. For these path models, each environmental raster has to be considered once as a potential resistance factor (i.e. impedes movement) and once as a potential conductance factor (i.e. as a variable that facilitates movement). In the context of the present study, we investigated the impact of the following environmental factors (Fig. 4): elevation, annual mean temperature, annual precipitation, key several land cover variables (e.g. “grasslands”, “savannas”, “forests”, “croplands”, “urban areas”; land cover categorized according to the International Geosphere Biosphere Program, IGBP), human population density, footprint [[7](#_ENREF_7)], major roads, and inaccessibility (time travel to nearest major cities of >50,000 inhabitants). The sources of the original raster files are listed in Table S2. Further, several distinct rasters were generated by transforming original raster cell values with the following formula: *v*_t_ = 1 + *k**(*v*_o_/*v*_max_), where *v*_t_ and *v*_o_ are the transformed and original raster cell values, and *v*_max_ the maximum raster cell value recorded in the raster. The rescaling parameter *k* here allows the definition and testing of different strengths of raster cell conductance or resistance, relative to the conductance/resistance of a cell with a minimum value set to “1”. For each of the three environmental factors, we tested three different values for *k* (i.e. *k* = 10, 100 and 1000).

*Step 3: estimating the correlation between branch durations and environmental distances.*

The correlation between the duration of each phylogeny branch and its environmental distance (see step 2) was estimated for each of the 100 posterior trees in the two data sets. Specifically, we estimated the statistic *Q* = R^2^_env_ - R^2^_null_, where R^2^_env_ is the coefficient of determination obtained when branch durations are regressed against environmental distances computed on the environmental raster, and R^2^_null_ is the coefficient of determination obtained when branch durations are regressed against environmental distances computed on the “null” raster, i.e. the environmental raster with a value of “1” assigned to all the cells (except cells with no original data). The *Q* statistic therefore represents how much variation in lineage movement is explained when spatial heterogeneity in the environmental variable is taken into account, above and beyond that explained by distance alone [[3](#_ENREF_3), [8](#_ENREF_8)]. Therefore, when *Q* > 0, distances weighted according to a heterogeneous environmental raster are correlated more strongly with branch duration than distances computed on a “null” raster (which represents geographical distance alone). Since one *Q* value was calculated per sampled posterior tree, we then obtained 100 *Q* values for each combination of environmental factor, *k* parameter value and path model. A variable can only be considered as potentially explanatory if both its distribution of regression coefficients and associated distribution of *Q* values are positive [[9](#_ENREF_9)]. Indeed, negative regression coefficients indicate that branch durations are negatively correlated with environmental distances and negative *Q* values indicate that considering environmental distances computed on the environmental raster rather than on the “null'' raster does not improve the linear regression fit. The statistical significance of a *Q* distribution was thus only tested (with the randomisation procedure of step 4) when at least 90% of the estimated *Q* values were positive.

*Step 4: testing statistical significance with a randomisation procedure.*

The statistical significance of the positive *Q* distributions was tested using a null model. To generate an appropriate null distribution for *Q*, we used a randomisation procedure [[1](#_ENREF_1), [3](#_ENREF_3)]: phylogenetic node positions were randomised within the study area, under the constraint that branch lengths, tree topology and root position are unchanged. Each sampled posterior tree was randomised once to generate null distributions of *Q* values that can be compared directly with the posterior distributions of estimated *Q* values. Each estimated *Q* value (*Q_estimated_*) was then compared to its corresponding randomised value (*Q_randomised_*) to compute a Bayes factor (BF). The BF support for a particular environmental factor was approximated by the posterior odds that *Q_estimated_* > *Q_randomised_* divided by the equivalent prior odds (the prior probability for *Q_estimated_* > *Q_randomised_* is considered to be 0.5):

$$\mathrm{BF}= {\frac{p_{e}}{1-p_{e}}}/{\frac{0.5}{1-0.5}}$$

where p_e_ is the posterior probability that *Q_estimated_* > *Q_randomised_*, i.e. the frequency at which *Q_estimated_* > *Q_randomised_* in the sampled posterior distribution. The prior odds is “1” because we have an equal prior expectation for *Q_estimated_* and *Q_randomised_*. The formal estimate of posterior predictive odds is analogous to computing Bayes Factors in case two alternative hypotheses exist, such for the inclusion of rate parameters or predictors in BSSVS procedures (Bayesian stochastic search variable selection; see equation [6] in Lemey *et al*. 2009 [[10](#_ENREF_10)]). As described in the scale of interpretation of BF’s defined by Kass & Raftery (1995) [[11](#_ENREF_11)], BF values higher than 3 and 20 can be respectively considered as “positive” and “strong” evidences of the statistical significance of *Q_estimated_*.

**Appendix S3. Analysis of the impact of environmental factor on RABV dispersal tendency in Yunnan**

In addition to the analyses based on lineage dispersal *velocity*, we also used a new analytical procedure to investigate the impact of several environmental factors on dispersal *tendency*. In this framework, environmental conditions are compared between nodes connected by a phylogeny branch. For each branch and environmental factor, we then computed the difference between the raster cell value at the oldest node position and at the youngest node position. These differences were then averaged within each sampled tree to obtain a single value *E*_estimated_ per tree, a value that is thus defined as the mean difference between the environmental values measured between two positions connected by a phylogeny branch. In a second step, we use the randomisation procedure described in Appendix S2 to obtain null distributions of environmental differences between oldest and youngest node positions. Each sampled posterior tree was randomised once to generate null distributions of *E* values. Each estimated *E* value (*E*_estimated_) was then compared to its corresponding randomised value (*E*_randomised_) to compute a Bayes factor (BF). For a given environmental factor tested as a potential positive driver of the dispersal, the BF support was approximated by the posterior odds that *E*_estimated_ < *E*_randomised_ divided by the equivalent prior odds (the prior probability for *E*_estimated_ < *E*_randomised_ is considered to be 0.5):

$$\mathrm{BF}= {\frac{p_{e}}{1-p_{e}}}/{\frac{0.5}{1-0.5}}$$

where *p_e_* is the posterior probability that *E*_estimated_ < *E*_randomised_, i.e. the frequency at which *E*_estimated_ < *E*_randomised_ in the sampled posterior distribution. The prior odds is 1 because we have an equal prior expectation for *E*_estimated_ and *E*_randomised_. When an environmental factor is tested as a potential positive driver of the dispersal, *p_e_* is instead defined as the posterior probability that *E*_estimated_ > *E*_randomised_. Following the same logic used for investigating the impact on dispersal velocity, each tested environmental factor was once tested as potential positive driver and once as a potential negative driver of the virus dispersal.

**References**

1. Dellicour S, Rose R, Faria NR, Lemey P, Pybus OG: **SERAPHIM: studying environmental rasters and phylogenetically-informed movements**. *Bioinformatics* 2016, **32**(20):3204-3206.

2. Pybus OG, Suchard MA, Lemey P, Bernardin FJ, Rambaut A, Crawford FW, Gray RR, Arinaminpathy N, Stramer SL, Busch MP: **Unifying the spatial epidemiology and molecular evolution of emerging epidemics**. *Proc Natl Acad Sci USA* 2012, **109**(37):15066-15071.

3. Dellicour S, Rose R, Pybus OG: **Explaining the geographic spread of emerging epidemics: a framework for comparing viral phylogenies and environmental landscape data**. *BMC bioinformatics* 2016, **17**(1):82.

4. Dijkstra EW: **A note on two problems in connexion with graphs**. *Numerische mathematik* 1959, **1**(1):269-271.

5. McRae BH: **Isolation by resistance**. *Evolution* 2006, **60**(8):1551-1561.

6. McRae BH, Dickson BG, Keitt TH, Shah VB: **Using circuit theory to model connectivity in ecology, evolution, and conservation**. *Ecology* 2008, **89**(10):2712-2724.

7. Wildlife Conservation Society - WCS, Center for International Earth Science Information Network - CIESIN - Columbia University: **Last of the Wild Project, Version 2, 2005 (LWP-2): Global Human Footprint Dataset (Geographic)**. In*.* Palisades, NY: NASA Socioeconomic Data and Applications Center (SEDAC); 2005.

8. Dellicour S, Rose R, Faria NR, Vieira LFP, Bourhy H, Gilbert M, Lemey P, Pybus OG: **Using viral gene sequences to compare and explain the heterogeneous spatial dynamics of virus epidemics**. *Mol Biol Evol* 2017:doi: 10.1093/molbev/msx1176.

9. Jacquot M, Nomikou K, Palmarini M, Mertens P, Biek R: **Bluetongue virus spread in Europe is a consequence of climatic, landscape and vertebrate host factors as revealed by phylogeographic inference**. *Proc Biol Sci* 2017, **284**(1864):20170919.

10. Lemey P, Rambaut A, Welch JJ, Suchard MA: **Phylogeography takes a relaxed random walk in continuous space and time**. *Mol Biol Evol* 2010, **27**(8):1877-1885.

11. Kass RE, Raftery AE: **Bayes factors**. *J Am Stat Assoc* 1995, **90**(430):773-795.

12. Gill MS, Lemey P, Bennett SN, Biek R, Suchard MA: **Understanding past population dynamics: Bayesian coalescent-based modeling with covariates**. *Syst Biol* 2016, **65**(6):1041-1056.

13. Trovão NS, Suchard MA, Baele G, Gilbert M, Lemey P: **Bayesian inference reveals host-specific contributions to the epidemic expansion of Influenza A H5N1**. *Mol Biol Evol* 2015, **32**(12):3264-3275.
