# Supplementary figures and images for: Transmission dynamics of re-emerging rabies in domestic dogs of rural China
Source: PLoS Pathog. 2018 Dec 6;14(12):e1007392. doi: 10.1371/journal.ppat.1007392 (PMC6283347; doi:10.1371/journal.ppat.1007392)

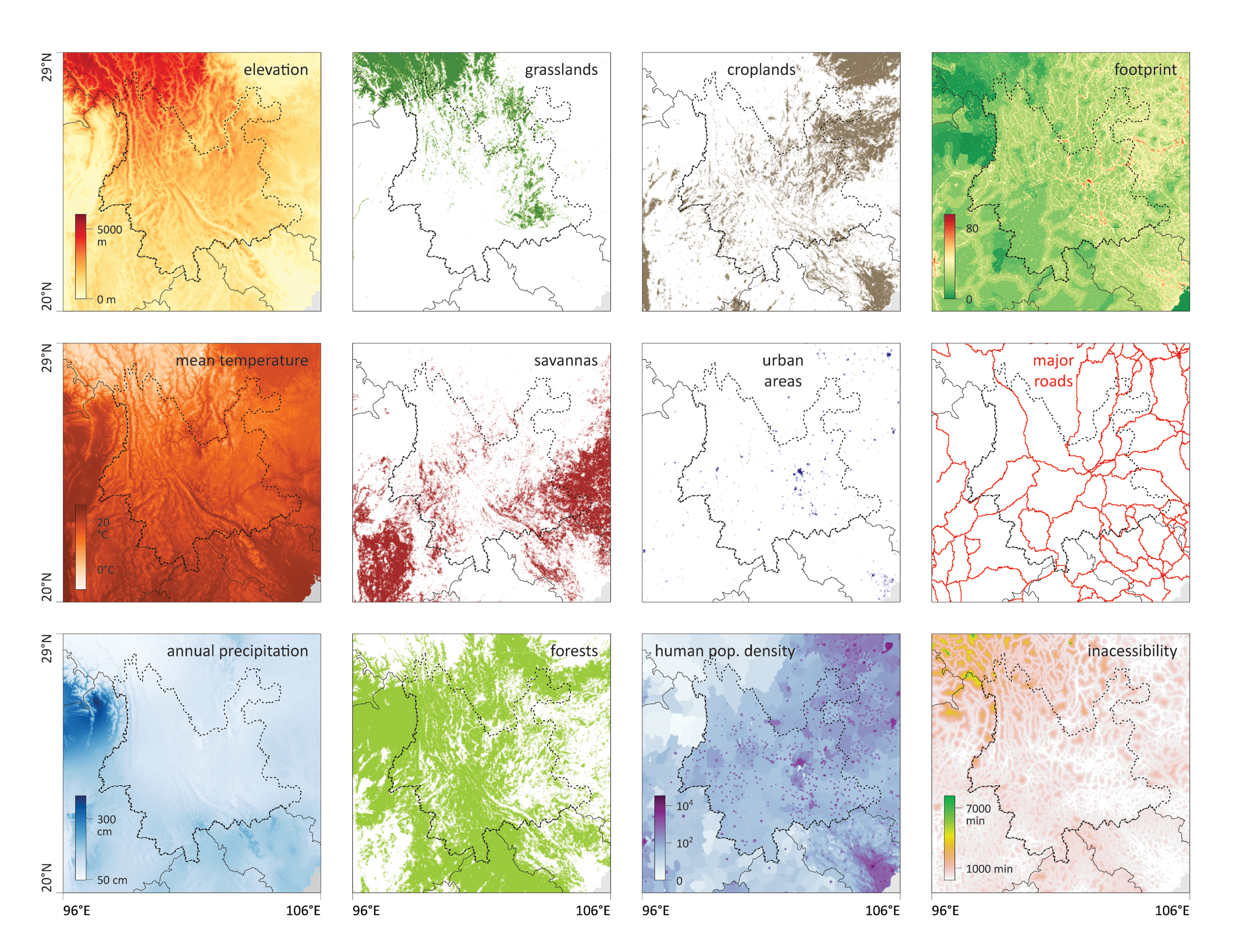

Supplement: S1 Fig — National and Yunnan province borders are respectively displayed by solid and dashed lines, respectively. (TIFF) [file ppat.1007392.s002.tiff]

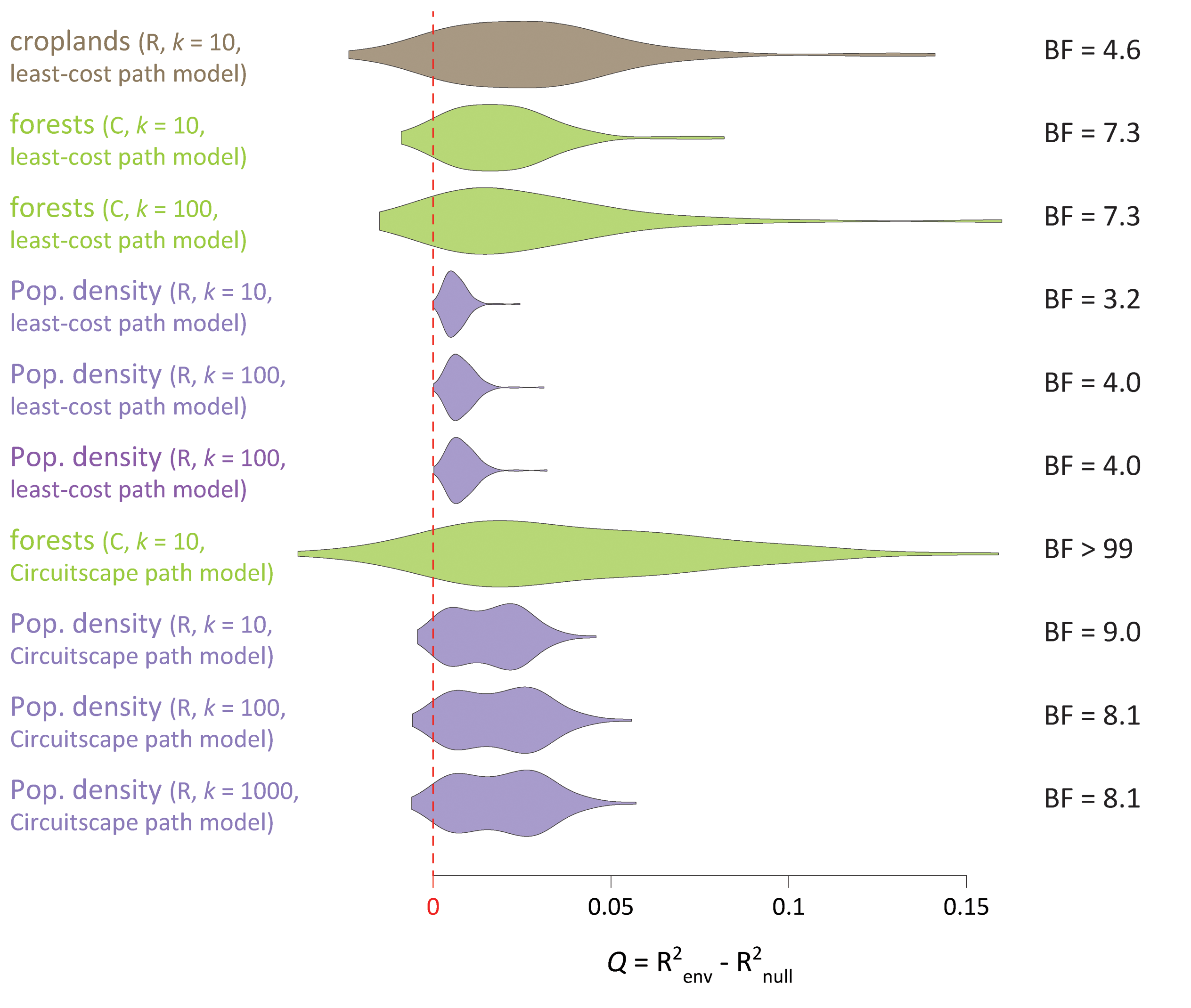

Supplement: S2 Fig — The results are based on 100 trees sampled from the posterior distribution. “C” and “R” indicate if the considered environmental variable was considered as a conductance ("C") or resistance factor ("R"), and k is the rescaling parameter used to transform the initial raster. Estimated Q distributions and related Bayes factor (BF) supports are reported here only for those model combinations with >90% of positive Q values and a BF support > 3 (see S3 Table for complete results). BF supports were estimated with the randomisation procedure detailed in Appendix S1 in S1 Text. Following Kass & Raftery (1995) we consider a BF > 3 and BF >20 respectively as a “positive” and “strong” evidences of the statistical significance of Q, i.e. the correlation between environmental distances and dispersal durations (see text for further details). (TIF) [file ppat.1007392.s003.tif]

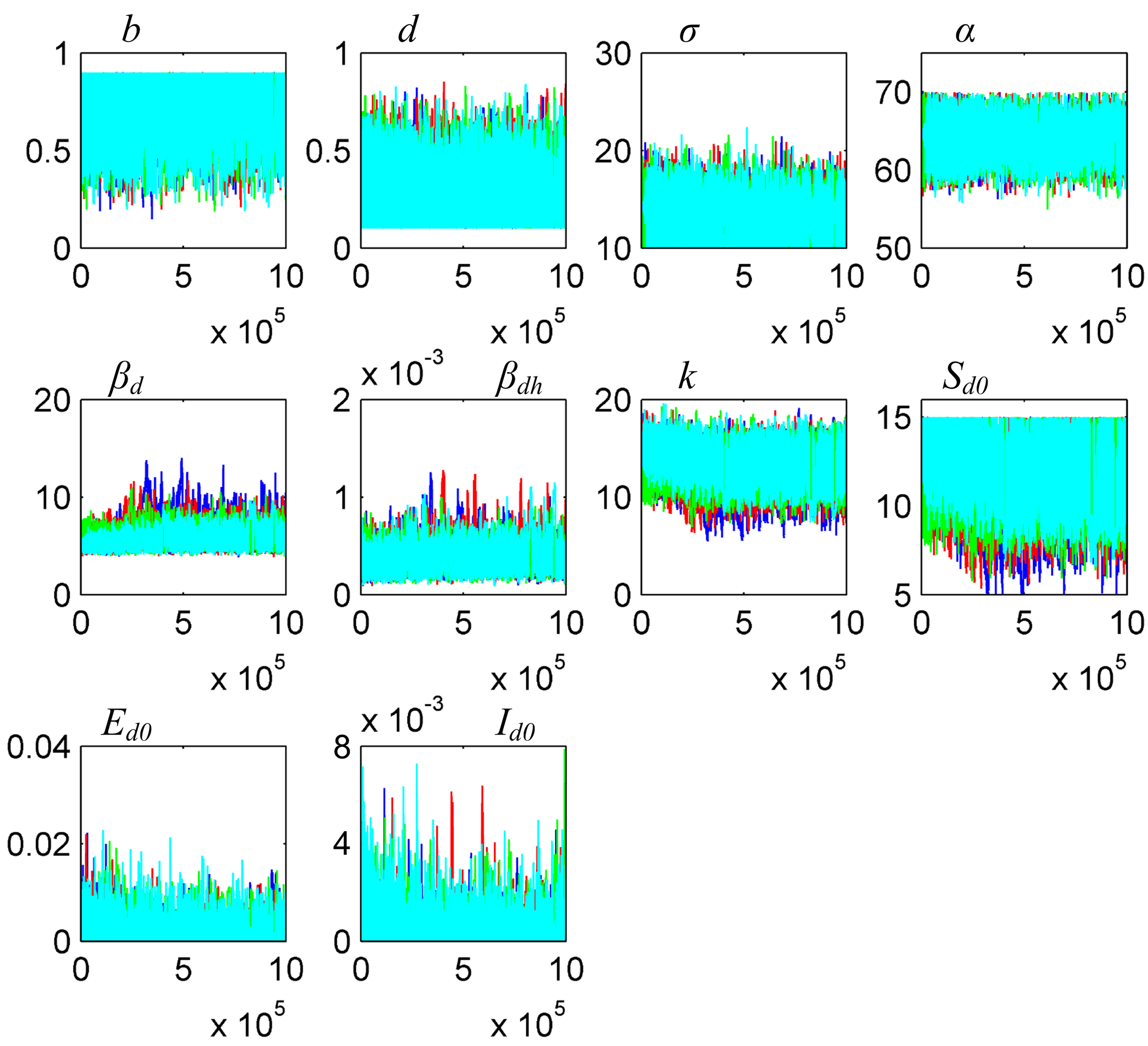

Supplement: S3 Fig — Four chains were initialized at different values, and they converge to the target distribution quickly. (TIF) [file ppat.1007392.s004.tif]

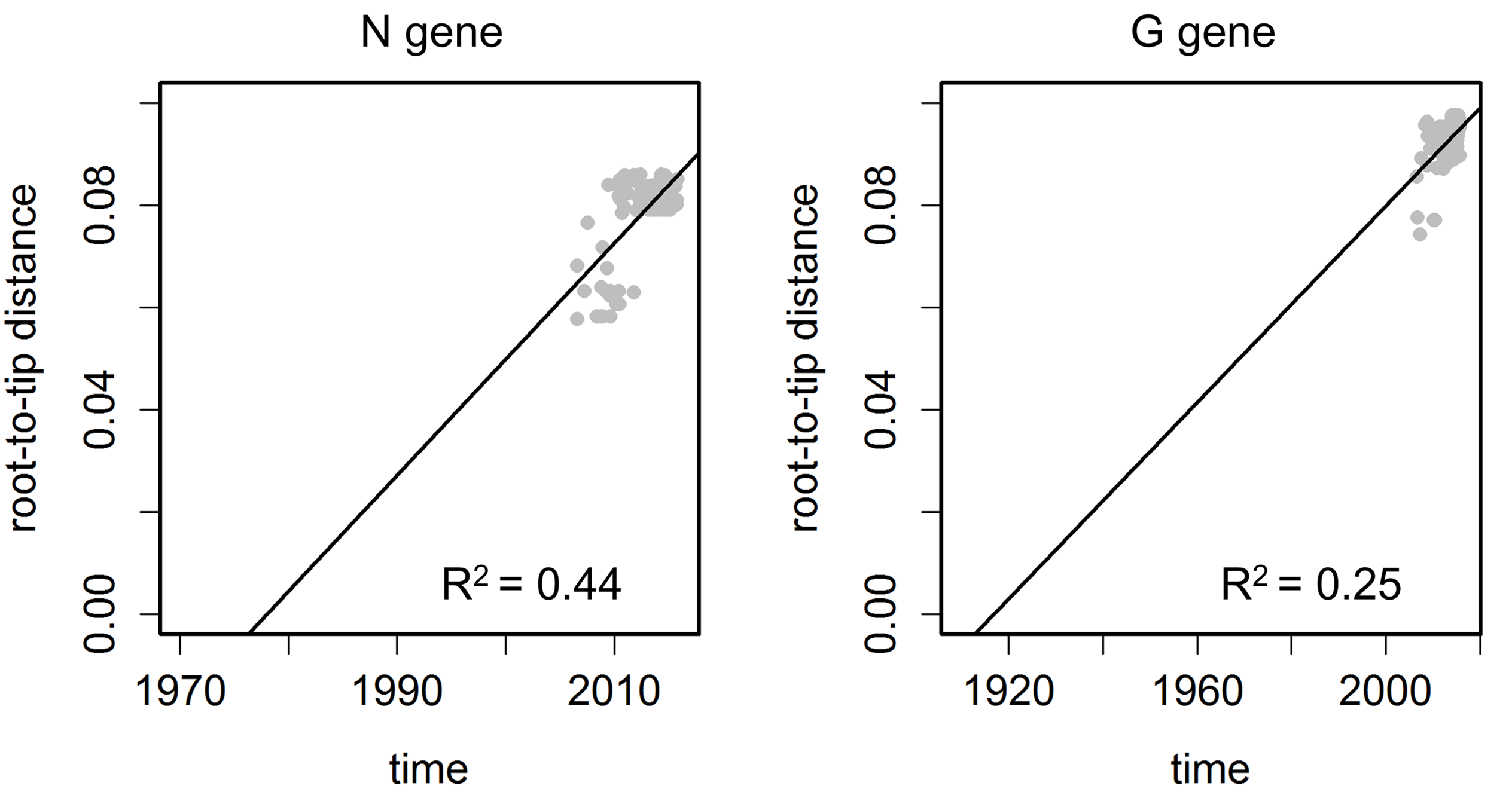

Supplement: S4 Fig — TempEst was used to obtain exploratory regressions based on the maximum likelihood trees. (TIF) [file ppat.1007392.s005.tif]
