# Supplementary material for: Transmission dynamics of re-emerging rabies in domestic dogs of rural China
Source: PLoS Pathog. 2018 Dec 6;14(12):e1007392. doi: 10.1371/journal.ppat.1007392 (PMC6283347; doi:10.1371/journal.ppat.1007392)
Supplement: S1 Table — (DOCX) [file ppat.1007392.s006.docx]

**S1 Table.** **List of RABV sequences analysed in this study.**

| Accession number | Year of isolation | Host of isolation | Sampling location | Gene |
| --- | --- | --- | --- | --- |
| KP072009 | 2012 | Dog | Yunnan | N |
| KP072010 | 2012 | Dog | Yunnan | N |
| KP072011 | 2012 | Dog | Yunnan | N |
| KP072012 | 2012 | Dog | Yunnan | N |
| KP072013 | 2012 | Dog | Yunnan | N |
| KP072014 | 2012 | Dog | Yunnan | N |
| KP072015 | 2012 | Dog | Yunnan | N |
| KP072016 | 2012 | Dog | Yunnan | N |
| KP072017 | 2012 | Human | Yunnan | N |
| KP072018 | 2012 | Dog | Yunnan | N |
| KP072019 | 2012 | Human | Yunnan | N |
| KP072020 | 2013 | Dog | Yunnan | N |
| KP072021 | 2013 | Dog | Yunnan | N |
| KP072022 | 2013 | Dog | Yunnan | N |
| KP072023 | 2013 | Dog | Yunnan | N |
| KP072024 | 2013 | Dog | Yunnan | N |
| KP072025 | 2013 | Dog | Yunnan | N |
| KP072026 | 2013 | Dog | Yunnan | N |
| KP072027 | 2013 | Dog | Yunnan | N |
| KP072028 | 2013 | Dog | Yunnan | N |
| KP072029 | 2013 | Dog | Yunnan | N |
| KP072030 | 2013 | Dog | Yunnan | N |
| KP202418 | 2013 | Dog | Yunnan | N |
| KP202419 | 2013 | Dog | Yunnan | N |
| KP202420 | 2013 | Dog | Yunnan | N |
| KP202421 | 2014 | Dog | Yunnan | N |
| KP202422 | 2014 | Dog | Yunnan | N |
| KP202423 | 2014 | Dog | Yunnan | N |
| KP202424 | 2014 | Dog | Yunnan | N |
| KP202425 | 2014 | Dog | Yunnan | N |
| KP202426 | 2014 | Dog | Yunnan | N |
| KP202427 | 2014 | Dog | Yunnan | N |
| KP202428 | 2014 | Dog | Yunnan | N |
| KP202429 | 2014 | Dog | Yunnan | N |
| KP202430 | 2014 | Dog | Yunnan | N |
| KP202431 | 2014 | Dog | Yunnan | N |
| KP202432 | 2014 | Dog | Yunnan | N |
| KP202433 | 2014 | Dog | Yunnan | N |
| KP202434 | 2014 | Dog | Yunnan | N |
| KP202435 | 2014 | Dog | Yunnan | N |
| KP202436 | 2014 | Dog | Yunnan | N |
| KP202437 | 2014 | Dog | Yunnan | N |
| KP202438 | 2014 | Dog | Yunnan | N |
| KP202439 | 2014 | Dog | Yunnan | N |
| KP202440 | 2014 | Human | Yunnan | N |
| KP202441 | 2014 | Dog | Yunnan | N |
| KP202442 | 2014 | Dog | Yunnan | N |
| KP202443 | 2014 | Dog | Yunnan | N |
| KP202444 | 2014 | Dog | Yunnan | N |
| KP202445 | 2014 | Dog | Yunnan | N |
| KP202446 | 2014 | Dog | Yunnan | N |
| KP202447 | 2014 | Dog | Yunnan | N |
| KP202448 | 2013 | Human | Yunnan | N |
| KT932670 | 2014 | Human | Yunnan | N |
| KT932671 | 2014 | Dog | Yunnan | N |
| KT932672 | 2014 | Dog | Yunnan | N |
| KT932673 | 2014 | Dog | Yunnan | N |
| KT932674 | 2014 | Dog | Yunnan | N |
| KT932675 | 2014 | Dog | Yunnan | N |
| KT932676 | 2014 | Dog | Yunnan | N |
| KT932677 | 2013 | Dog | Yunnan | N |
| KT932678 | 2013 | Dog | Yunnan | N |
| KT932679 | 2013 | Dog | Yunnan | N |
| KT932680 | 2013 | Dog | Yunnan | N |
| KT932681 | 2013 | Dog | Yunnan | N |
| KT932682 | 2014 | Dog | Yunnan | N |
| KT932683 | 2014 | Dog | Yunnan | N |
| KT932684 | 2014 | Dog | Yunnan | N |
| KT932685 | 2014 | Dog | Yunnan | N |
| KT932686 | 2015 | Dog | Yunnan | N |
| KT932687 | 2015 | Dog | Yunnan | N |
| KT932688 | 2015 | Dog | Yunnan | N |
| KT932689 | 2015 | Dog | Yunnan | N |
| KT932690 | 2015 | Dog | Yunnan | N |
| KT932691 | 2015 | Dog | Yunnan | N |
| KT932692 | 2015 | Human | Yunnan | N |
| KT932693 | 2015 | Dog | Yunnan | N |
| KT932694 | 2015 | Dog | Yunnan | N |
| KT932695 | 2015 | Dog | Yunnan | N |
| KT932696 | 2015 | Dog | Yunnan | N |
| KT932697 | 2015 | Dog | Yunnan | N |
| KT932698 | 2015 | Dog | Yunnan | N |
| KX096992 | 2015 | Dog | Yunnan | N |
| KX096993 | 2015 | Dog | Yunnan | N |
| KX096994 | 2015 | Dog | Yunnan | N |
| KX096995 | 2015 | Dog | Yunnan | N |
| KX096996 | 2015 | Dog | Yunnan | N |
| KX096997 | 2015 | Dog | Yunnan | N |
| KX096998 | 2015 | Dog | Yunnan | N |
| KX096999 | 2015 | Cattle | Yunnan | N |
| KX097000 | 2015 | Dog | Yunnan | N |
| JF819597 | 2010 | Dog | Yunnan | G |
| JF819598 | 2010 | Human | Yunnan | G |
| JF819599 | 2010 | Dog | Yunnan | G |
| JF819600 | 2010 | Dog | Yunnan | G |
| JF819601 | 2010 | cattle | Yunnan | G |
| JF819602 | 2010 | Dog | Yunnan | G |
| JQ040570 | 2011 | Dog | Yunnan | G |
| JQ040571 | 2010 | Dog | Yunnan | G |
| JQ040572 | 2008 | Dog | Yunnan | G |
| JQ040573 | 2008 | Dog | Yunnan | G |
| JQ040574 | 2010 | Human | Yunnan | G |
| JQ040575 | 2010 | Dog | Yunnan | G |
| JQ040576 | 2010 | Dog | Yunnan | G |
| JQ040577 | 2008 | Dog | Yunnan | G |
| JQ040578 | 2009 | Dog | Yunnan | G |
| JQ040579 | 2008 | Dog | Yunnan | G |
| JQ040580 | 2011 | Dog | Yunnan | G |
| JQ040581 | 2011 | Dog | Yunnan | G |
| JX276383 | 2010 | Dog | Yunnan | G |
| JX276384 | 2012 | Dog | Yunnan | G |
| JX276385 | 2012 | Dog | Yunnan | G |
| JX276386 | 2012 | Dog | Yunnan | G |
| JX276387 | 2012 | Dog | Yunnan | G |
| JX276388 | 2012 | Dog | Yunnan | G |
| JX276389 | 2012 | Dog | Yunnan | G |
| JX276390 | 2012 | Dog | Yunnan | G |
| JX276391 | 2012 | Dog | Yunnan | G |
| JX276392 | 2012 | Dog | Yunnan | G |
| JX276393 | 2012 | Dog | Yunnan | G |
| JX276394 | 2012 | Dog | Yunnan | G |
| JX276395 | 2012 | Dog | Yunnan | G |
| JX276396 | 2011 | Dog | Yunnan | G |
| JX276397 | 2010 | Dog | Yunnan | G |
| JX276398 | 2011 | Dog | Yunnan | G |
| JX276399 | 2011 | Dog | Yunnan | G |
| JX276400 | 2012 | Dog | Yunnan | G |
| JX276401 | 2012 | Dog | Yunnan | G |
| JX276402 | 2010 | Dog | Yunnan | G |
| JX276403 | 2012 | Human | Yunnan | G |
| JX276404 | 2012 | Dog | Yunnan | G |
| KP072031 | 2012 | Dog | Yunnan | G |
| KP072032 | 2012 | Dog | Yunnan | G |
| KP072033 | 2012 | Dog | Yunnan | G |
| KP072034 | 2012 | Dog | Yunnan | G |
| KP072035 | 2012 | Dog | Yunnan | G |
| KP072036 | 2012 | Dog | Yunnan | G |
| KP072037 | 2012 | Dog | Yunnan | G |
| KP072038 | 2012 | Human | Yunnan | G |
| KP072039 | 2012 | Dog | Yunnan | G |
| KP072040 | 2012 | Human | Yunnan | G |
| KP072041 | 2013 | Dog | Yunnan | G |
| KP072042 | 2013 | Dog | Yunnan | G |
| KP072043 | 2013 | Dog | Yunnan | G |
| KP072044 | 2013 | Dog | Yunnan | G |
| KP072045 | 2013 | Dog | Yunnan | G |
| KP072046 | 2013 | Dog | Yunnan | G |
| KP072047 | 2013 | Dog | Yunnan | G |
| KP072048 | 2013 | Dog | Yunnan | G |
| KP072049 | 2013 | Dog | Yunnan | G |
| KP072050 | 2013 | Dog | Yunnan | G |
| KP072051 | 2013 | Dog | Yunnan | G |
| KP072052 | 2013 | Dog | Yunnan | G |
| KP202402 | 2014 | Dog | Yunnan | G |
| KP202403 | 2014 | Dog | Yunnan | G |
| KP202404 | 2014 | Dog | Yunnan | G |
| KP202405 | 2014 | Dog | Yunnan | G |
| KP202406 | 2014 | Dog | Yunnan | G |
| KP202407 | 2014 | Dog | Yunnan | G |
| KP202408 | 2014 | Dog | Yunnan | G |
| KP202409 | 2014 | Dog | Yunnan | G |
| KP202410 | 2014 | Dog | Yunnan | G |
| KP202411 | 2014 | Dog | Yunnan | G |
| KP202412 | 2014 | Dog | Yunnan | G |
| KP202413 | 2014 | Human | Yunnan | G |
| KP202414 | 2014 | Dog | Yunnan | G |
| KP202415 | 2014 | Dog | Yunnan | G |
| KP202416 | 2014 | Dog | Yunnan | G |
| KP202417 | 2014 | Dog | Yunnan | G |
| KT861554 | 2013 | Dog | Yunnan | G |
| KT861555 | 2014 | Dog | Yunnan | G |
| KT861556 | 2014 | Dog | Yunnan | G |
| KT861557 | 2014 | Dog | Yunnan | G |
| KT861558 | 2014 | Dog | Yunnan | G |
| KT861559 | 2014 | Human | Yunnan | G |
| KT861560 | 2014 | Dog | Yunnan | G |
| KT861561 | 2014 | Dog | Yunnan | G |
| KT861562 | 2014 | Dog | Yunnan | G |
| KT861563 | 2014 | Dog | Yunnan | G |
| KT861564 | 2014 | Dog | Yunnan | G |
| KT861565 | 2014 | Dog | Yunnan | G |
| KT861566 | 2014 | Dog | Yunnan | G |
| KT861567 | 2014 | Dog | Yunnan | G |
| KT861568 | 2014 | Dog | Yunnan | G |
| KT861569 | 2014 | Dog | Yunnan | G |
| KT861570 | 2014 | Dog | Yunnan | G |
| KT861571 | 2014 | Dog | Yunnan | G |
| KT861572 | 2014 | Dog | Yunnan | G |
| KT861573 | 2014 | Dog | Yunnan | G |
| KT861574 | 2015 | Dog | Yunnan | G |
| KT861575 | 2015 | Dog | Yunnan | G |
| KT861576 | 2015 | Dog | Yunnan | G |
| KT861577 | 2015 | Dog | Yunnan | G |
| KT861578 | 2015 | Dog | Yunnan | G |
| KT861579 | 2015 | Human | Yunnan | G |
| KT861580 | 2015 | Dog | Yunnan | G |
| KT861581 | 2015 | Dog | Yunnan | G |
| KT861582 | 2015 | Dog | Yunnan | G |
| KT861583 | 2015 | Dog | Yunnan | G |
| KT861584 | 2015 | Dog | Yunnan | G |
| KT861585 | 2015 | Dog | Yunnan | G |
| KT861586 | 2015 | Dog | Yunnan | G |
| KX096983 | 2015 | Dog | Yunnan | G |
| KX096984 | 2015 | Dog | Yunnan | G |
| KX096985 | 2015 | Dog | Yunnan | G |
| KX096986 | 2015 | Dog | Yunnan | G |
| KX096987 | 2015 | Dog | Yunnan | G |
| KX096988 | 2015 | Dog | Yunnan | G |
| KX096989 | 2015 | Dog | Yunnan | G |
| KX096990 | 2015 | Cattle | Yunnan | G |
| KX096991 | 2015 | Dog | Yunnan | G |
