# Supplementary material for: Transmission dynamics of re-emerging rabies in domestic dogs of rural China
Source: PLoS Pathog. 2018 Dec 6;14(12):e1007392. doi: 10.1371/journal.ppat.1007392 (PMC6283347; doi:10.1371/journal.ppat.1007392)
Supplement: S2 Table — The results are based on 100 trees sampled in each posterior distribution. “C” and “R” indicate if the considered environmental raster was considered as a conductance ("C") or resistance factor ("R"), and k is the rescaling parameter used to transform the initial raster (see the Appendix S2 in S1 Text for further details). For regression coefficients and Q values we report both the median estimate and the 95% HPD interval. The Bayes factor (BF) supports based on the randomisation procedure is only reported when p(Q > 0) is at least 90%. Following Kass & Raftery (1995) we consider a Bayes factor (BF) >3 as positive support for a significant correlation between the environmental distances and dispersal durations. (DOCX) [file ppat.1007392.s007.docx]

**S2 Table. Impact of several environmental factors on RABV dispersal velocity.** The results are based on 100 trees sampled in each posterior distribution. “C” and “R” indicate if the considered environmental raster was considered as a conductance ("C") or resistance factor ("R"), and *k* is the rescaling parameter used to transform the initial raster (see the Appendix S2 for further details). For regression coefficients and *Q* values we report both the median estimate and the 95% HPD interval. The Bayes factor (BF) supports based on the randomisation procedure is only reported when *p*(*Q* > 0) is at least 90%. Following Kass & Raftery (1995) we consider a Bayes factor (BF) >3 as positive support for a significant correlation between the environmental distances and dispersal durations.

| **Path model** | **Environmental factor** | ***k*** | **Regression coefficient** | ***Q* statistic** | ***p*(*Q* > 0)** | **BF** |
| --- | --- | --- | --- | --- | --- | --- |
| least-cost | accessibility (C) | 10 | 0.035 [0.018, 2.226] | 0.001 [-0.010, 0.010] | 0.59 | - |
|  |  | 100 | 0.143 [0.074, 9.177] | 0.003 [-0.022, 0.025] | 0.58 | - |
|  |  | 1000 | 1.081 [0.562, 73.121] | 0.000 [-0.029, 0.029] | 0.47 | - |
|  | accessibility (R) | 10 | 0.017 [0.008, 0.996] | 0.000 [-0.007, 0.004] | 0.45 | - |
|  |  | 100 | 0.006 [0.003, 0.293] | -0.003 [-0.025, 0.010] | 0.32 | - |
|  |  | 1000 | 0.001 [0.000, 0.034] | -0.007 [-0.033, 0.009] | 0.22 | - |
|  | annual mean | 10 | 0.158 [0.079, 9.882] | 0.004 [-0.002, 0.016] | 0.88 | - |
|  | temperature (C) | 100 | 1.386 [0.699, 86.926] | 0.005 [-0.003, 0.019] | 0.88 | - |
|  |  | 1000 | 13.661 [6.900, 857.295] | 0.005 [-0.003, 0.019] | 0.88 | - |
|  | annual mean | 10 | 0.003 [0.002, 0.184] | -0.004 [-0.016, 0.001] | 0.06 | - |
|  | temperature (R) | 100 | 0.000 [0.000, 0.021] | -0.005 [-0.018, 0.001] | 0.06 | - |
|  |  | 1000 | 0.000 [0.000, 0.002] | -0.005 [-0.018, 0.001] | 0.06 | - |
|  | annual | 10 | 0.083 [0.042, 5.498] | 0.005 [0.001, 0.013] | 0.99 | 1.5 |
|  | precipitation (C) | 100 | 0.640 [0.328, 42.870] | 0.007 [0.001, 0.016] | 0.99 | 2.3 |
|  |  | 1000 | 6.206 [3.182, 416.385] | 0.007 [0.001, 0.017] | 0.99 | 2.3 |
|  | annual | 10 | 0.005 [0.003, 0.316] | -0.005 [-0.014, 0.000] | 0.02 | - |
|  | precipitation (R) | 100 | 0.001 [0.000, 0.040] | -0.007 [-0.018, -0.001] | 0.02 | - |
|  |  | 1000 | 0.000 [0.000, 0.004] | -0.007 [-0.019, -0.001] | 0.02 | - |
|  | croplands (C) | 10 | 0.043 [0.019, 2.720] | -0.019 [-0.045, -0.005] | 0.00 | - |
|  |  | 100 | 0.056 [-0.473, 5.133] | -0.033 [-0.073, -0.005] | 0.00 | - |
|  |  | 1000 | 0.021 [-1.780, 5.912] | -0.039 [-0.081, -0.004] | 0.02 | - |
|  | croplands (R) | 10 | 0.019 [0.010, 1.067] | 0.025 [-0.013, 0.101] | 0.92 | 4.6 |
|  |  | 100 | 0.006 [0.003, 0.391] | 0.038 [-0.047, 0.175] | 0.78 | - |
|  |  | 1000 | 0.001 [0.000, 0.045] | 0.026 [-0.062, 0.246] | 0.73 | - |
|  | elevation (C) | 10 | 0.091 [0.044, 5.304] | -0.003 [-0.012, 0.002] | 0.07 | - |
|  |  | 100 | 0.677 [0.349, 40.323] | -0.004 [-0.015, 0.002] | 0.05 | - |
|  |  | 1000 | 6.559 [3.387, 390.226] | -0.004 [-0.016, 0.002] | 0.05 | - |
|  | elevation (R) | 10 | 0.006 [0.003, 0.392] | 0.004 [-0.004, 0.012] | 0.87 | - |
|  |  | 100 | 0.001 [0.000, 0.053] | 0.004 [-0.007, 0.016] | 0.82 | - |
|  |  | 1000 | 0.000 [0.000, 0.005] | 0.004 [-0.007, 0.016] | 0.81 | - |
|  | foot print (C) | 10 | 0.079 [0.040, 4.656] | -0.003 [-0.020, 0.003] | 0.15 | - |
|  |  | 100 | 0.598 [0.300, 33.918] | -0.005 [-0.025, 0.003] | 0.13 | - |
|  |  | 1000 | 5.787 [2.906, 326.289] | -0.005 [-0.026, 0.003] | 0.13 | - |
|  | foot print (R) | 10 | 0.007 [0.003, 0.436] | 0.005 [-0.004, 0.019] | 0.91 | 1.8 |
|  |  | 100 | 0.001 [0.000, 0.062] | 0.006 [-0.006, 0.024] | 0.90 | - |
|  |  | 1000 | 0.000 [0.000, 0.007] | 0.006 [-0.006, 0.025] | 0.89 | - |
|  | forests (C) | 10 | 0.222 [0.113, 13.671] | 0.017 [-0.004, 0.054] | 0.93 | 7.3 |
|  |  | 100 | 1.849 [0.874, 100.127] | 0.02 [-0.009, 0.104] | 0.91 | 7.3 |
|  |  | 1000 | 4.423 [0.819, 171.143] | -0.023 [-0.069, 0.101] | 0.09 | - |
|  | forests (R) | 10 | 0.003 [0.001, 0.172] | -0.021 [-0.049, -0.002] | 0.00 | - |
|  |  | 100 | 0.000 [-0.001, 0.016] | -0.027 [-0.063, -0.005] | 0.00 | - |
|  |  | 1000 | 0.000 [0.000, 0.001] | -0.028 [-0.065, -0.005] | 0.01 | - |
|  | grasslands (C) | 10 | 0.025 [0.014, 1.156] | -0.013 [-0.041, 0.005] | 0.07 | - |
|  |  | 100 | 0.027 [-0.130, 1.103] | -0.02 [-0.061, 0.008] | 0.08 | - |
|  |  | 1000 | 0.028 [-0.202, 0.903] | -0.022 [-0.063, 0.008] | 0.07 | - |
|  | grasslands (R) | 10 | 0.021 [0.010, 1.295] | 0.003 [-0.002, 0.017] | 0.83 | - |
|  |  | 100 | 0.017 [0.008, 1.045] | 0.01 [-0.016, 0.087] | 0.79 | - |
|  |  | 1000 | 0.004 [0.001, 0.310] | -0.013 [-0.060, 0.250] | 0.30 | - |
|  | human pop. | 10 | 0.112 [0.053, 6.522] | -0.003 [-0.010, 0.000] | 0.03 | - |
|  | density (C) | 100 | 0.912 [0.431, 52.828] | -0.004 [-0.012, 0.000] | 0.03 | - |
|  |  | 1000 | 8.910 [4.210, 515.837] | -0.004 [-0.013, 0.000] | 0.03 | - |
|  | human pop. | 10 | 0.005 [0.003, 0.322] | 0.006 [0.002, 0.013] | 0.99 | 3.2 |
|  | density (R) | 100 | 0.001 [0.000, 0.041] | 0.007 [0.003, 0.016] | 1.00 | 4.0 |
|  |  | 1000 | 0.000 [0.000, 0.004] | 0.007 [0.003, 0.016] | 1.00 | 4.0 |
|  | major roads (C) | 10 | 0.085 [0.044, 2.766] | 0.012 [-0.041, 0.062] | 0.71 | - |
|  |  | 100 | 0.097 [0.038, 3.951] | 0.002 [-0.052, 0.068] | 0.54 | - |
|  |  | 1000 | 0.097 [0.016, 4.050] | 0.000 [-0.054, 0.063] | 0.50 | - |
|  | major roads (R) | 10 | 0.017 [0.008, 1.044] | -0.002 [-0.012, 0.008] | 0.34 | - |
|  |  | 100 | 0.005 [0.002, 0.290] | -0.011 [-0.047, 0.019] | 0.21 | - |
|  |  | 1000 | 0.001 [-0.002, 0.029] | -0.016 [-0.061, 0.021] | 0.14 | - |
|  | savannas (C) | 10 | 0.048 [0.027, 2.673] | 0.010 [-0.012, 0.030] | 0.87 | - |
|  |  | 100 | 0.102 [0.058, 4.951] | 0.009 [-0.032, 0.055] | 0.73 | - |
|  |  | 1000 | 0.126 [0.062, 5.492] | 0.002 [-0.047, 0.066] | 0.54 | - |
|  | savannas (R) | 10 | 0.019 [0.009, 1.231] | -0.001 [-0.012, 0.007] | 0.29 | - |
|  |  | 100 | 0.011 [0.005, 1.287] | -0.009 [-0.043, 0.024] | 0.16 | - |
|  |  | 1000 | 0.001 [-0.003, 0.051] | -0.031 [-0.078, 0.005] | 0.03 | - |
|  | urban areas (C) | 10 | 0.026 [0.013, 1.432] | 0.002 [-0.009, 0.010] | 0.77 | - |
|  |  | 100 | 0.030 [0.015, 1.530] | 0.004 [-0.014, 0.016] | 0.74 | - |
|  |  | 1000 | 0.031 [0.016, 1.560] | 0.004 [-0.016, 0.017] | 0.74 | - |
|  | urban areas (R) | 10 | 0.022 [0.010, 1.324] | 0.000 [0.000, 0.003] | 0.58 | - |
|  |  | 100 | 0.021 [0.010, 1.309] | 0.000 [-0.004, 0.012] | 0.36 | - |
|  |  | 1000 | 0.009 [0.001, 0.661] | -0.023 [-0.056, 0.040] | 0.05 | - |
| Circuitscape | accessibility (C) | 10 | 1.825 [1.220, 60.212] | 0.000 [-0.010, 0.017] | 0.52 | - |
|  |  | 100 | 3.664 [2.119, 160.526] | -0.027 [-0.072, 0.009] | 0.08 | - |
|  |  | 1000 | 8.275 [2.708, 422.745] | -0.064 [-0.131, 0.004] | 0.04 | - |
|  | accessibility (R) | 10 | 0.831 [0.508, 22.289] | -0.007 [-0.025, 0.003] | 0.13 | - |
|  |  | 100 | 0.162 [0.090, 4.224] | -0.031 [-0.068, 0.007] | 0.06 | - |
|  |  | 1000 | 0.017 [0.009, 0.481] | -0.040 [-0.084, 0.008] | 0.05 | - |
|  | annual mean | 10 | 8.986 [5.622, 287.278] | 0.007 [-0.015, 0.024] | 0.81 | - |
|  | temperature (C) | 100 | 78.185 [48.015, 2522.743] | 0.007 [-0.018, 0.026] | 0.81 | - |
|  |  | 1000 | 769.96 [471.802, 24870.042] | 0.007 [-0.018, 0.026] | 0.81 | - |
|  | annual mean | 10 | 0.170 [0.109, 4.907] | -0.010 [-0.033, 0.002] | 0.05 | - |
|  | temperature (R) | 100 | 0.019 [0.012, 0.550] | -0.011 [-0.036, 0.002] | 0.05 | - |
|  |  | 1000 | 0.002 [0.001, 0.056] | -0.012 [-0.036, 0.002] | 0.04 | - |
|  | annual | 10 | 5.106 [3.337, 159.467] | 0.008 [-0.005, 0.031] | 0.94 | 1.9 |
|  | precipitation (C) | 100 | 39.435 [25.350, 1214.477] | 0.01 [-0.008, 0.035] | 0.95 | 1.9 |
|  |  | 1000 | 382.425 [244.848, 11754.947] | 0.01 [-0.009, 0.036] | 0.95 | 1.9 |
|  | annual | 10 | 0.285 [0.179, 8.141] | -0.017 [-0.043, -0.002] | 0.02 | - |
|  | precipitation (R) | 100 | 0.034 [0.022, 0.978] | -0.023 [-0.051, -0.002] | 0.01 | - |
|  |  | 1000 | 0.004 [0.002, 0.100] | -0.024 [-0.052, -0.003] | 0.00 | - |
|  | croplands (C) | 10 | 0.880 [-0.078, 28.603] | -0.061 [-0.104, -0.004] | 0.01 | - |
|  |  | 100 | 0.274 [-11.007, 32.683] | -0.079 [-0.136, -0.004] | 0.01 | - |
|  |  | 1000 | -0.102 [-19.022, 36.840] | -0.079 [-0.141, -0.004] | 0.01 | - |
|  | croplands (R) | 10 | 0.452 [0.268, 20.917] | 0.034 [-0.030, 0.106] | 0.86 | - |
|  |  | 100 | 0.050 [0.026, 2.587] | 0.003 [-0.084, 0.095] | 0.60 | - |
|  |  | 1000 | 0.005 [0.002, 0.256] | -0.004 [-0.096, 0.090] | 0.38 | - |
|  | elevation (C) | 10 | 4.409 [2.793, 122.259] | -0.011 [-0.027, -0.001] | 0.01 | - |
|  |  | 100 | 30.356 [19.104, 830.858] | -0.019 [-0.042, -0.002] | 0.01 | - |
|  |  | 1000 | 289.156 [181.722, 7898.242] | -0.020 [-0.044, -0.002] | 0.01 | - |
|  | elevation (R) | 10 | 0.319 [0.195, 9.814] | 0.001 [-0.016, 0.010] | 0.55 | - |
|  |  | 100 | 0.040 [0.024, 1.271] | -0.001 [-0.022, 0.011] | 0.47 | - |
|  |  | 1000 | 0.004 [0.002, 0.131] | -0.001 [-0.023, 0.011] | 0.47 | - |
|  | foot print (C) | 10 | 3.998 [2.297, 118.376] | -0.014 [-0.036, 0.000] | 0.04 | - |
|  |  | 100 | 27.611 [15.559, 813.459] | -0.021 [-0.044, 0.000] | 0.03 | - |
|  |  | 1000 | 263.421 [148.05, 7772.479] | -0.022 [-0.045, 0.000] | 0.03 | - |
|  | foot print (R) | 10 | 0.375 [0.232, 11.751] | 0.006 [-0.011, 0.031] | 0.81 | - |
|  |  | 100 | 0.049 [0.031, 1.585] | 0.007 [-0.013, 0.035] | 0.72 | - |
|  |  | 1000 | 0.005 [0.003, 0.164] | 0.007 [-0.014, 0.036] | 0.71 | - |
|  | forests (C) | 10 | 6.945 [4.037, 290.633] | 0.032 [-0.016, 0.110] | 0.93 | >99 |
|  |  | 100 | 17.181 [5.436, 546.735] | -0.013 [-0.088, 0.150] | 0.31 | - |
|  |  | 1000 | 11.368 [1.003, 307.19] | -0.072 [-0.134, 0.079] | 0.06 | - |
|  | forests (R) | 10 | 0.083 [-0.087, 2.439] | -0.052 [-0.090, -0.006] | 0.00 | - |
|  |  | 100 | 0.007 [-0.059, 0.223] | -0.062 [-0.107, -0.006] | 0.00 | - |
|  |  | 1000 | 0.001 [-0.007, 0.021] | -0.063 [-0.110, -0.006] | 0.00 | - |
|  | grasslands (C) | 10 | 1.227 [0.711, 30.081] | -0.016 [-0.045, -0.003] | 0.01 | - |
|  |  | 100 | 0.93 [-9.049, 21.351] | -0.037 [-0.081, -0.006] | 0.00 | - |
|  |  | 1000 | 0.775 [-13.098, 17.268] | -0.046 [-0.099, -0.004] | 0.00 | - |
|  | grasslands (R) | 10 | 0.936 [0.543, 36.354] | 0.002 [-0.030, 0.086] | 0.61 | - |
|  |  | 100 | 0.146 [0.054, 10.195] | -0.037 [-0.097, 0.146] | 0.24 | - |
|  |  | 1000 | 0.011 [0.002, 0.999] | -0.057 [-0.121, 0.152] | 0.14 | - |
|  | human pop. | 10 | 5.518 [3.354, 145.558] | -0.018 [-0.031, -0.001] | 0.00 | - |
|  | density (C) | 100 | 42.974 [26.004, 1111.968] | -0.022 [-0.038, -0.001] | 0.00 | - |
|  |  | 1000 | 417.446 [252.484, 10788.969] | -0.022 [-0.039, -0.001] | 0.00 | - |
|  | human pop. | 10 | 0.300 [0.193, 8.857] | 0.016 [0.000, 0.033] | 0.97 | 9.0 |
|  | density (R) | 100 | 0.037 [0.024, 1.113] | 0.019 [0.000, 0.041] | 0.97 | 8.1 |
|  |  | 1000 | 0.004 [0.002, 0.114] | 0.019 [0.000, 0.042] | 0.97 | 8.1 |
|  | major roads (C) | 10 | 1.485 [0.878, 43.885] | -0.002 [-0.026, 0.015] | 0.42 | - |
|  |  | 100 | 1.515 [0.819, 43.187] | -0.012 [-0.055, 0.015] | 0.19 | - |
|  |  | 1000 | 1.423 [0.339, 40.713] | -0.020 [-0.069, 0.009] | 0.17 | - |
|  | major roads (R) | 10 | 0.435 [0.210, 13.919] | -0.046 [-0.09, 0.002] | 0.04 | - |
|  |  | 100 | 0.014 [-0.178, 0.828] | -0.077 [-0.137, -0.002] | 0.02 | - |
|  |  | 1000 | 0.000 [-0.043, 0.060] | -0.078 [-0.140, -0.003] | 0.02 | - |
|  | savannas (C) | 10 | 1.573 [0.934, 46.960] | -0.005 [-0.028, 0.007] | 0.31 | - |
|  |  | 100 | 1.494 [0.789, 42.461] | -0.029 [-0.084, 0.009] | 0.13 | - |
|  |  | 1000 | 1.158 [0.265, 41.307] | -0.047 [-0.119, 0.007] | 0.07 | - |
|  | savannas (R) | 10 | 0.690 [0.375, 32.95] | -0.018 [-0.061, 0.029] | 0.09 | - |
|  |  | 100 | 0.053 [-0.016, 2.370] | -0.066 [-0.125, 0.026] | 0.04 | - |
|  |  | 1000 | 0.002 [-0.046, 0.247] | -0.073 [-0.136, 0.018] | 0.04 | - |
|  | urban areas (C) | 10 | 1.351 [0.837, 39.188] | -0.001 [-0.012, 0.002] | 0.43 | - |
|  |  | 100 | 1.374 [0.797, 41.005] | -0.004 [-0.022, 0.004] | 0.36 | - |
|  |  | 1000 | 1.376 [0.783, 41.629] | -0.005 [-0.027, 0.004] | 0.34 | - |
|  | urban areas (R) | 10 | 1.197 [0.723, 34.209] | -0.002 [-0.013, 0.028] | 0.22 | - |
|  |  | 100 | 0.320 [0.062, 10.280] | -0.054 [-0.111, 0.034] | 0.05 | - |
|  |  | 1000 | 0.002 [-0.088, 0.066] | -0.074 [-0.142, -0.005] | 0.02 | - |
