# Supplementary material for: Transmission dynamics of re-emerging rabies in domestic dogs of rural China
Source: PLoS Pathog. 2018 Dec 6;14(12):e1007392. doi: 10.1371/journal.ppat.1007392 (PMC6283347; doi:10.1371/journal.ppat.1007392)
Supplement: S3 Table — (DOCX) [file ppat.1007392.s008.docx]

S3 Table. Source of data for each environmental raster.

| **Original raster** | **Source** | **URL** |
| --- | --- | --- |
| Land cover | IGBP (International Geosphere Biosphere Program) | www.igbp.net |
| Elevation | SRTM (Shuttle Radar Topography Mission) | webmap.ornl.gov |
| Human density | MAP (Malaria Atlas Project) | www.map.ox.ac.uk |
| Inaccessibility | Joint Research Centre of the European Commission | bioval.jr.ec.europea.eu |
| Annual mean temperature | Bioclimatic variable of the WordClim database | www.worldclim.org/ |
| Annual precipitation | Bioclimatic variable of the WordClim database | www.worldclim.org/ |
| Major roads | NIMA (National Imagery and Mapping Agency's) | www.mapability.com |
| Footprint | WCS (Wildlife Conservation Society) | sedac.ciesin.columbia.edu |
