# Supplementary material for: Transmission dynamics of re-emerging rabies in domestic dogs of rural China
Source: PLoS Pathog. 2018 Dec 6;14(12):e1007392. doi: 10.1371/journal.ppat.1007392 (PMC6283347; doi:10.1371/journal.ppat.1007392)
Supplement: S4 Table — For model parameters, we used the same range as for the prior distribution. 1000 parameter sets were sampled with latin hypercube sampling, and partial rank correlation coefficients were estimated. (DOCX) [file ppat.1007392.s009.docx]

**S4 Table. Sensitivity analysis.** For model parameters, we used the same range as for the prior distribution. 1000 parameter sets were sampled with latin hypercube sampling, and partial rank correlation coefficients were estimated.

| Model parameters | Distribution | Peak intensity | Peak date |
| --- | --- | --- | --- |
| *b_d_* | Uniform[0,1] | -0.09* | -0.02 |
| *d_d_* | Uniform[0,1] | 0.04 | 0.07* |
| *σ_d_* | Uniform[10,30] | 0.08* | -0.02 |
| *α_d_* | Uniform[40,70] | -0.32* | -0.05* |
| *β_d_* | Uniform[0,10] | 0.78* | -0.01 |
| *β_dh_* | Uniform[0,10] | 0.79* | -0.03 |
| *K* | Uniform[5,15] | -0.11* | -0.08* |
| Initial conditions |  |  |  |
| *S_d0_* | Uniform[5,10] | 0.58* | -0.01 |
| *E_d0_* | Uniform[0,1] | 0.29* | -0.10* |
| *I_d0_* | Uniform[0,1] | 0.29* | -0.05* |

* *P* < 0.05
